# Electrochemically Triggered Co-Conformational Switching in a [2]catenane Comprising a Non-Symmetric Calix[6]arene Wheel and a Two-Station Oriented Macrocycle

**DOI:** 10.3390/molecules23051156

**Published:** 2018-05-11

**Authors:** Valeria Zanichelli, Luca Dallacasagrande, Arturo Arduini, Andrea Secchi, Giulio Ragazzon, Serena Silvi, Alberto Credi

**Affiliations:** 1 Dipartimento di Scienze Chimiche, della Vita e della Sostenibilità Ambientale, Università di Parma, Parco Area delle Scienze 17/A, 43124 Parma, Italy; valeria.zanichelli@studenti.unipr.it (V.Z.); luca.dallacasagrande@studenti.unipr.it (L.D.); arturo.arduini@unipr.it (A.A.); 2 Dipartimento di Chimica “G. Ciamician”, Università di Bologna, 40126 Bologna, Italy; giulio.ragazzon@unibo.it (G.R.); serena.silvi@unibo.it (S.S.); 3 Dipartimento di Scienze Chimiche, Università di Padova, 35131 Padova, Italy; 4 Center for Light Activated Nanostructures (CLAN), Università di Bologna and Consiglio Nazionale delle Ricerche, 40129 Bologna, Italy; 5 Dipartimento di Scienze e Tecnologie Agro-alimentari, Università di Bologna, 40127 Bologna, Italy; 6 Istituto per la Sintesi Organica e la Fotoreattività, Consiglio Nazionale delle Ricerche, 40129 Bologna, Italy

**Keywords:** bipyridinium, calix[6]arenes, catenanes, electrochemistry, molecular machines, synthesis, ring-closing metathesis, voltammetry

## Abstract

Catenanes with desymmetrized ring components can undergo co-conformational rearrangements upon external stimulation and can form the basis for the development of molecular rotary motors. We describe the design, synthesis and properties of a [2]catenane consisting of a macrocycle—the ‘track’ ring—endowed with two distinct recognition sites (a bipyridinium and an ammonium) for a calix[6]arene—the ‘shuttle’ ring. By exploiting the ability of the calixarene to thread appropriate non-symmetric axles with directional selectivity, we assembled an oriented pseudorotaxane and converted it into the corresponding oriented catenane by intramolecular ring closing metathesis. Cyclic voltammetric experiments indicate that the calixarene wheel initially surrounds the bipyridinium site, moves away from it when it is reduced, and returns in the original position upon reoxidation. A comparison with appropriate model compounds shows that the presence of the ammonium station is necessary for the calixarene to leave the reduced bipyridinium site.

## 1. Introduction

The chemistry of catenanes—i.e., mechanically interlocked molecules (MIMs) consisting of two or more entwined macrocycles [1,2,3]—has lately experienced a remarkable development, in particular thanks to improved synthetic protocols [4] that disclosed the realization of molecular architectures of increasing complexity. The first synthetic attempts to obtain these interlocked molecules relied on a statistical approach [5] that, unfortunately, yielded negligible amounts of the target catenanes. More recently, the progresses in template-directed syntheses provided new, more elegant and better performing strategies in terms of accessible structures and reaction yields. Several classes of macrocycles have been employed as the rings, and a wide set of non-covalent interactions, such as electron donor-acceptor interactions [6,7], hydrogen bonding [8,9], metal ion coordination [10,11], and anion binding [12], has been exploited to gather the components into preorganized assemblies that can undergo catenation in a predictable and tunable way.

**Figure 1:**
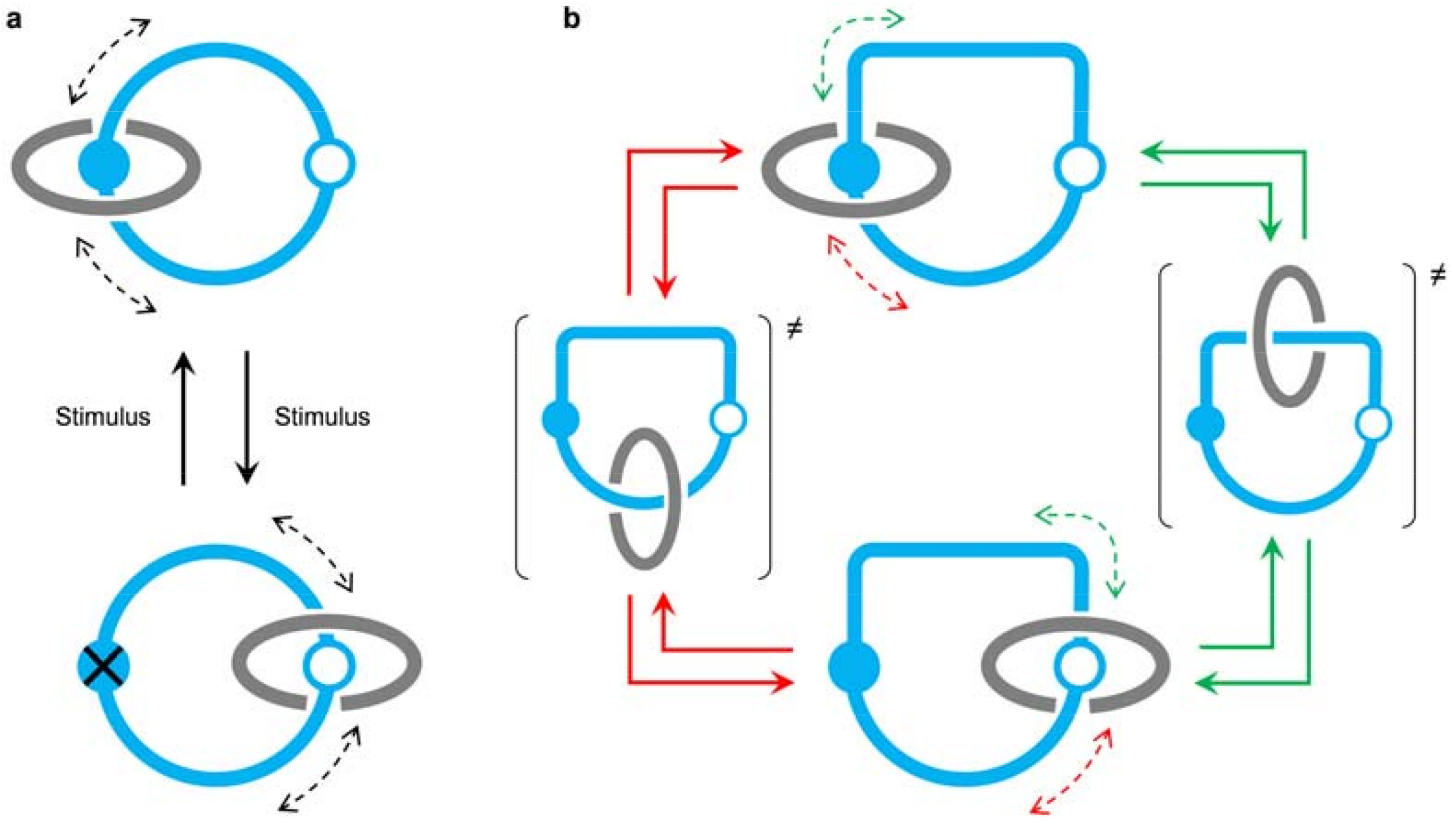
(**a**) In a [2]catenane containing two different recognition sites on one ring, the relative rotation of the rings is achieved upon switching off and on the primary recognition site with an external stimulus. As the clockwise and anticlockwise directions are probable equally, no repetitive unidirectional circumrotation can be obtained. (**b**) In a bistable [2]catenane with an oriented ‘track’ ring, the clockwise and anticlockwise rotation directions are not equivalent. This is a key requirement for the construction of catenane rotary motors.

An interesting behavior of properly designed catenanes is the ability of the rings to rotate with respect to one another, that is, to behave as molecular machines [2,13,14]. To accomplish this task, specific functional units have to be incorporated into the macrocycles so that controlled co-conformational rearrangements can be triggered by appropriate stimuli. The most common design involves the presence on one ring (the ‘track’) of two different recognition sites for the other ring (the ‘shuttle’); upon switching off and on the affinity of the primary site, the ‘shuttle’ ring moves onto and away from the secondary one, and reversible pirouetting can be achieved (Figure 1a) [15,16,17,18,19].

Much more interesting are catenanes that exhibit unidirectional circumrotation—that is, a full rotation of one ring relative to the other in either clockwise or anticlockwise direction—because they form the basis for the construction of molecular rotary motors [2,13,14]. Following the approach shown in Figure 1a, directional rotary motion requires that the clockwise and anticlockwise half-rotations are non-equivalent and correlated, so that full rotation in a direction is always faster than in the other. Such a result was cleverly obtained by Leigh and coworkers by exploiting a series of orthogonal chemical transformations [20] and queuing effects in [3]catenanes [21]. More recently, unidirectional ring rotation in catenanes was obtained with a single chemical reactant [22,23]. As shown by all these examples, a general requirement for unidirectional rotation is that the ‘track’ ring be oriented, that is, its macrocyclic structure travelled in a given direction is not equivalent to that in the other direction (Figure 1b).

**Figure 2:**
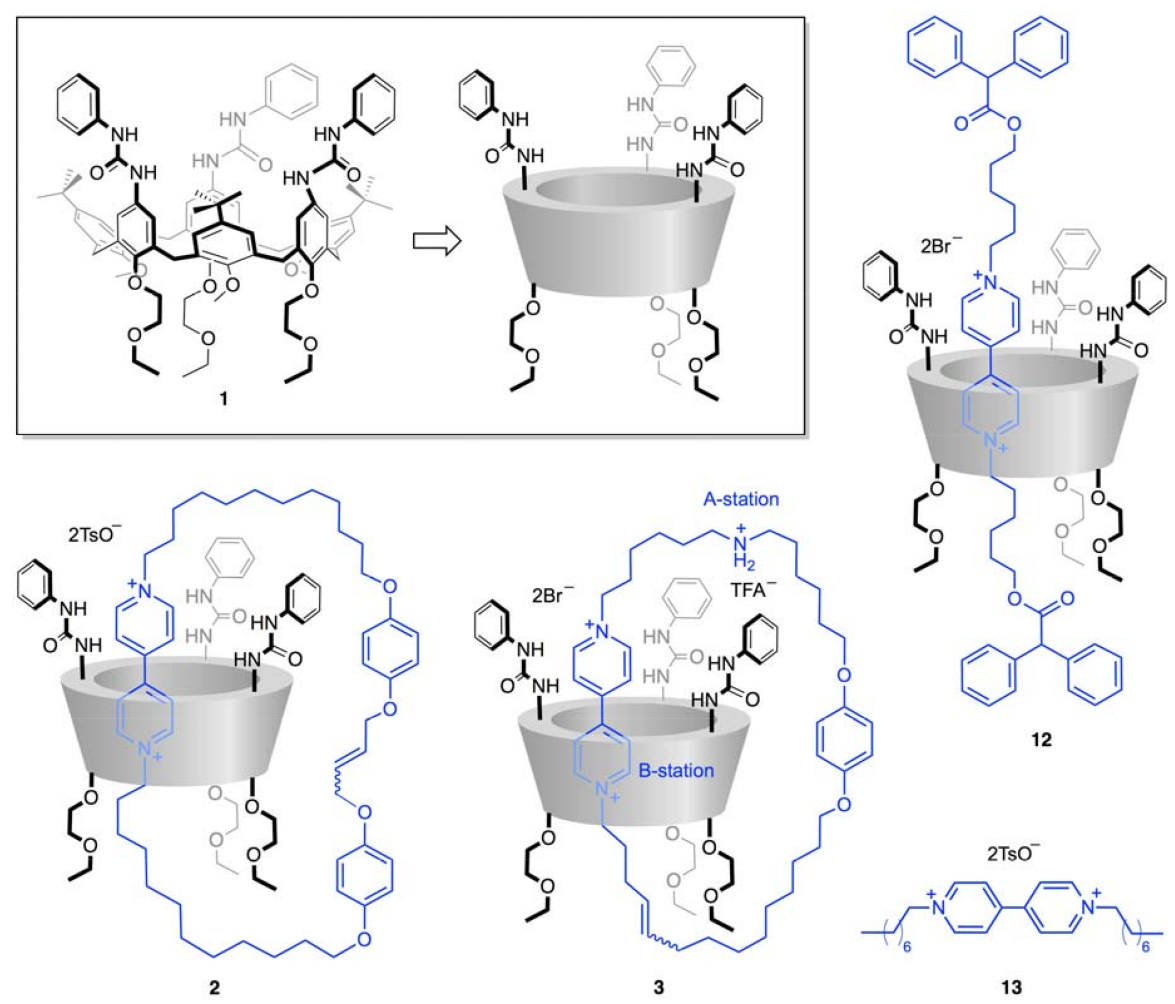
Structural formula of the investigated compounds and model species: the tris(*N*-phenylureido)calix[6]arene-based wheel **1** and its cartoon representation, the one-station [2]catenane **2**, the two-station oriented [2]catenane **3**, the [2]rotaxane **12**, and the bipyridinium-based axle **13**.

Calix[6]arenes possess a cavity large enough to be pierced by aromatic moieties; therefore, they can be employed as ring components in catenanes. The first calix[6]arene [2]catenane was reported in 2013 by Neri and coworkers [24]. The catenation step consisted in the reaction of the two terminal OH groups of a preformed calix[6]arene-based pseudorotaxane complex with an α,ω-diisocyanate. More recently, we used the tris(*N*-phenylureido)calix[6]arene wheel **1** to synthesize [2]catenane **2** (Figure 2), by applying as the catenation step an intramolecular ring-closing metathesis reaction (RCM) on the terminal allylic groups of the axial component of a pre-assembled pseudorotaxane [25]. The latter is stabilized by a plethora of weak intermolecular interactions between **1** and the 4,4′-bipyridinium central unit of the axle [26,27].

Building upon this work, and in view of our interest in the development of novel stimuli-responsive molecular machines [26,28], we report on the synthesis and electrochemical properties of the calix[6]arene-based [2]catenane **3** (Figure 2). This compound consists of wheel **1** and an interlocked macrocyclic ring endowed with two recognition sites for the calixarene, namely, a 4,4′-bipyridinium (B-station) and a secondary ammonium (A-station) units. The position of these stations along the macrocycle render it oriented, remarkably because of the chemically distinct rims of the three-dimensional wheel **1**; catenane **3** is one of two structural isomers that differ in the orientation of the calixarene with respect to the other ring. As the B-station is redox-active, and its interaction with **1** is frustrated upon reduction [29], we aim at understanding whether the electrochemical stimulation of **3** can cause co-conformational changes [30]. Although the development of a rotary motor is beyond the scope of the present study, the desymmetrized structure of catenanes such as **3** makes them appealing for the study of the rotation direction.

## 2. Results and Discussion

### 2.1. Synthesis and Characterization of Calix[6]arene-Based [2]catenanes

**Scheme 1:**
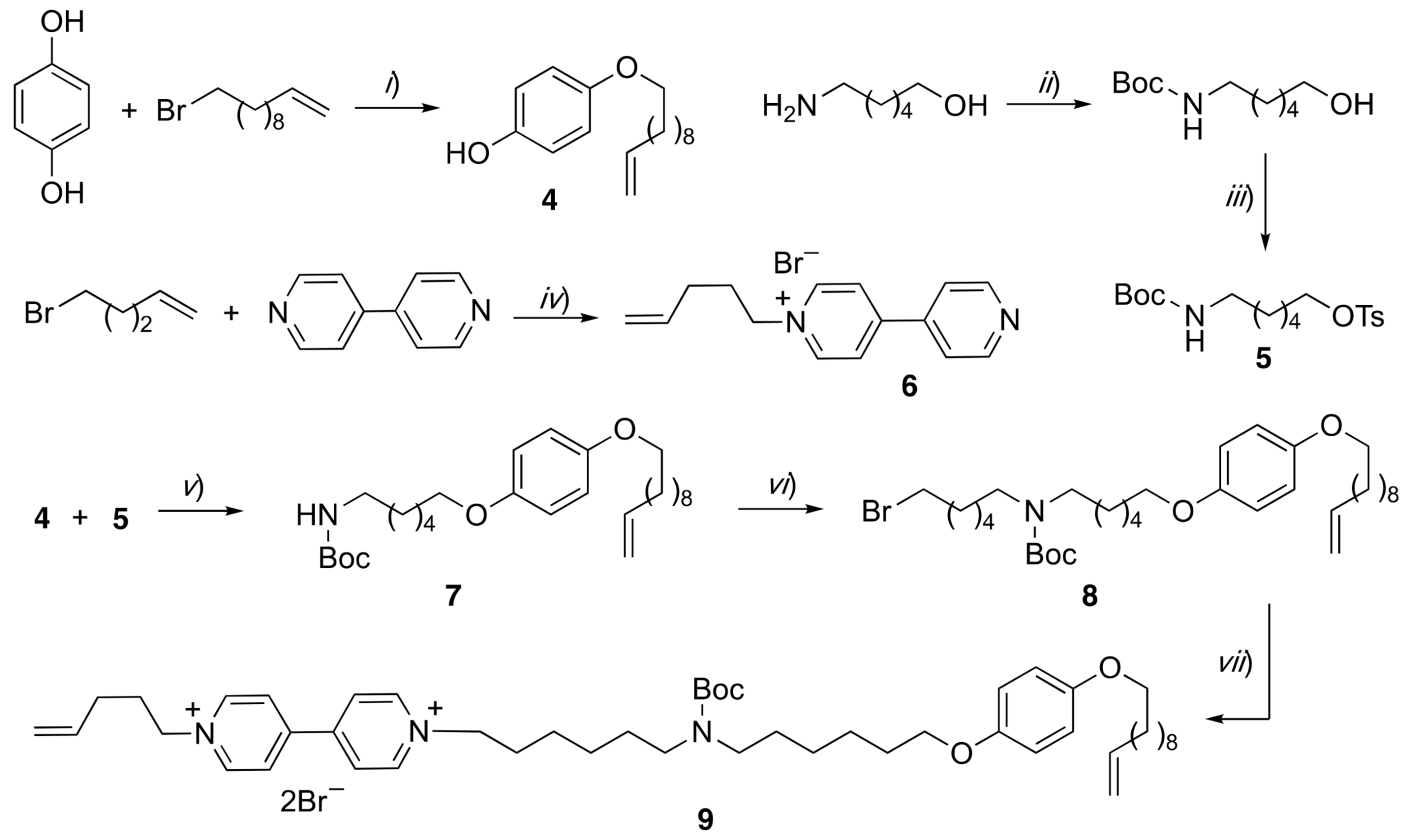
Reagents and conditions: (i) K_2_CO_3_, *N*,*N*-dimethylformamide (DMF), 80 °C, 48 h, 65%; (ii) Boc_2_O, MeOH, triethylamine, reflux, 1 h, 95%; (iii) TsCl, 4-(dimethylamino)pyridine (DMAP), triethylamine, CH_2_Cl_2_, rt, 48 h, 81%; (iv) CH_3_CN, 80 °C, 24 h, 80%; (v) K_2_CO_3_, DMF, 80 °C, 18 h, 58%; (vi) 1,6-dibromohexane, NaH, DMF, rt, 14 h, 50%; (vii) **6**, CH_3_CN, reflux, 7 days, 55%.

**Scheme 2:**
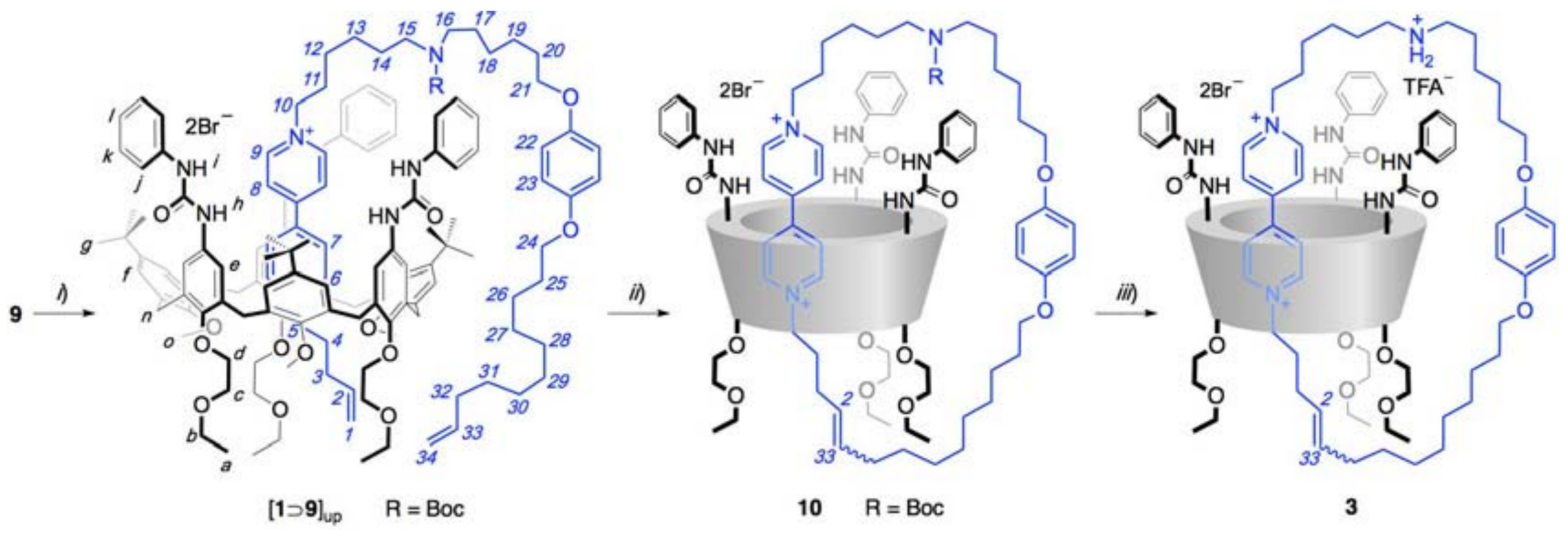
Reagents and conditions: (i) **1**, toluene, rt, 12 h, quant.; (ii) 2nd gen Grubbs cat., toluene, rt, 48 h, 17%; (iii) Trifluoroacetic acid (TFA), CH_2_Cl_2_, rt, 3 h, quant.

**Figure 3:**
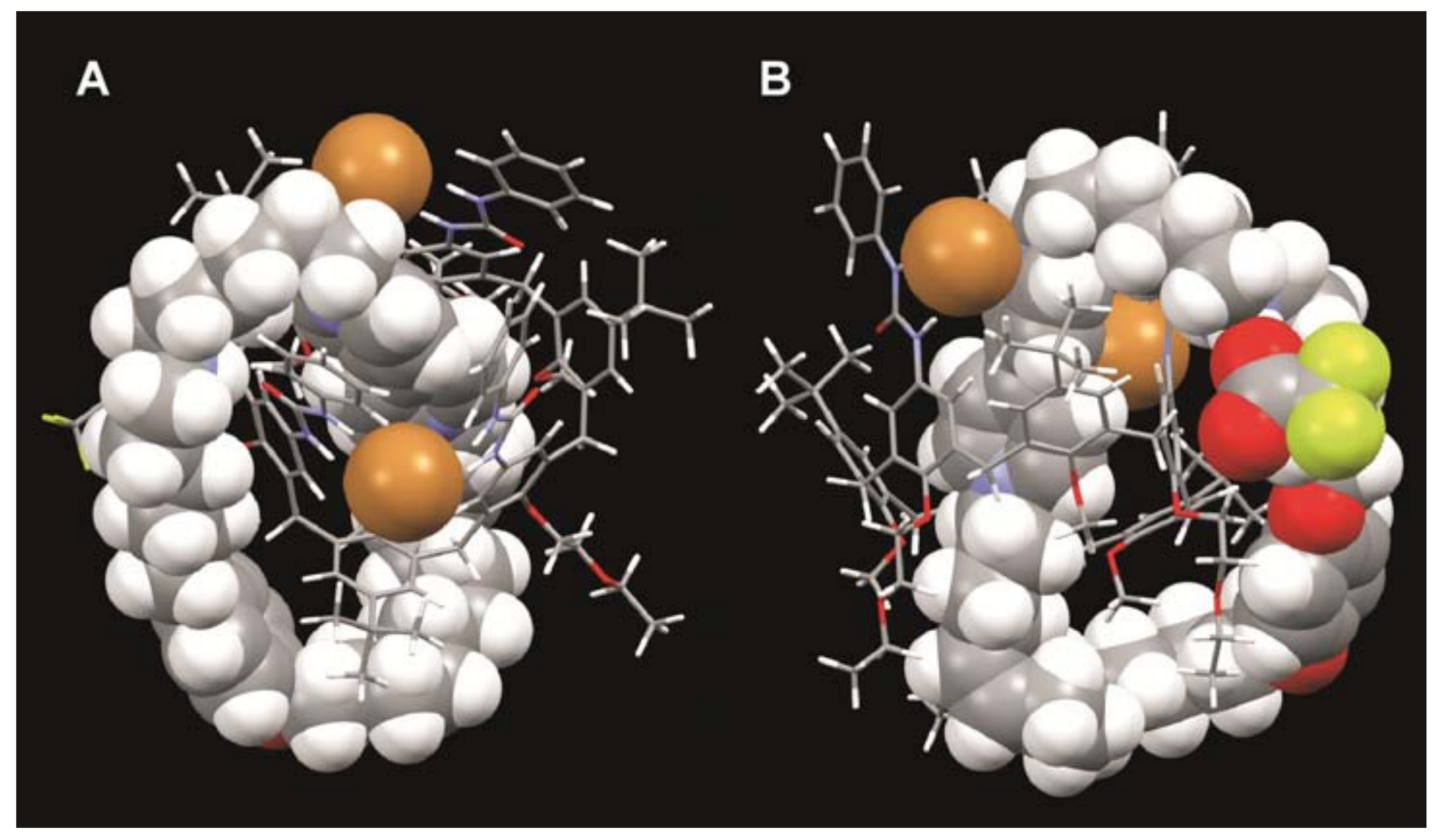
Two perspective views of the minimized structure (molecular mechanics, MMFF94s force field) of [2]catenane **3**. To appreciate the features of the interlocked structure, the calix[6]arene wheel and the bipyridinium-containing macrocycle (together with the anions) are rendered with the stick and the space-filling modes, respectively.

For the synthesis of the oriented [2]catenane **3** (Figure 2), we adopted the approach previously used for the preparation of the non-oriented [2]catenane **2** [25]. Specifically, we synthesized the bipyridinium-based axle **9** (Scheme 1), endowed with two ω-alkenyl chains of different lengths. The longest (30 atoms) of the two chains was designed to embed the A-station as a secondary amine group protected with a *tert*-butyloxycarbonyl (Boc) protecting group. The other chain was chosen premeditatedly short (5 atoms) to promote the directional threading of that extremity of the axle from the upper rim of the calix[6]arene. In line with previous observations [31], the oriented pseudorotaxane [**1**⊃**9**]_up_, in which the A-station is located near the upper calixarene rim, is expected (Scheme 2). We used molecular mechanics calculations (Figure 3) to identify the overall ideal length of the axle to enable the intramolecular RCM step while minimizing possible intermolecular oligomerization side reactions.

The oriented pseudorotaxane [**1**⊃**9**]_up_ was assembled by equilibrating axle **9** with an equimolar amount of wheel **1** in toluene at room temperature (Scheme 2). Indeed, as previously stated [31], under such conditions, **9** selectively threads **1** from its upper rim with the shorter C5 alkenyl chain, since the longer and functionalized side chain acts as a kinetic stopper. The structure and the relative arrangement of the thread with respect the rims of the calix[6]arene macrocycle in [**1**⊃**9**]_up_ were confirmed by a series of Nuclear Magnetic Resonance (NMR) measurements (see Figures S3–S6 of the Supplementary Material).

**Figure 4:**
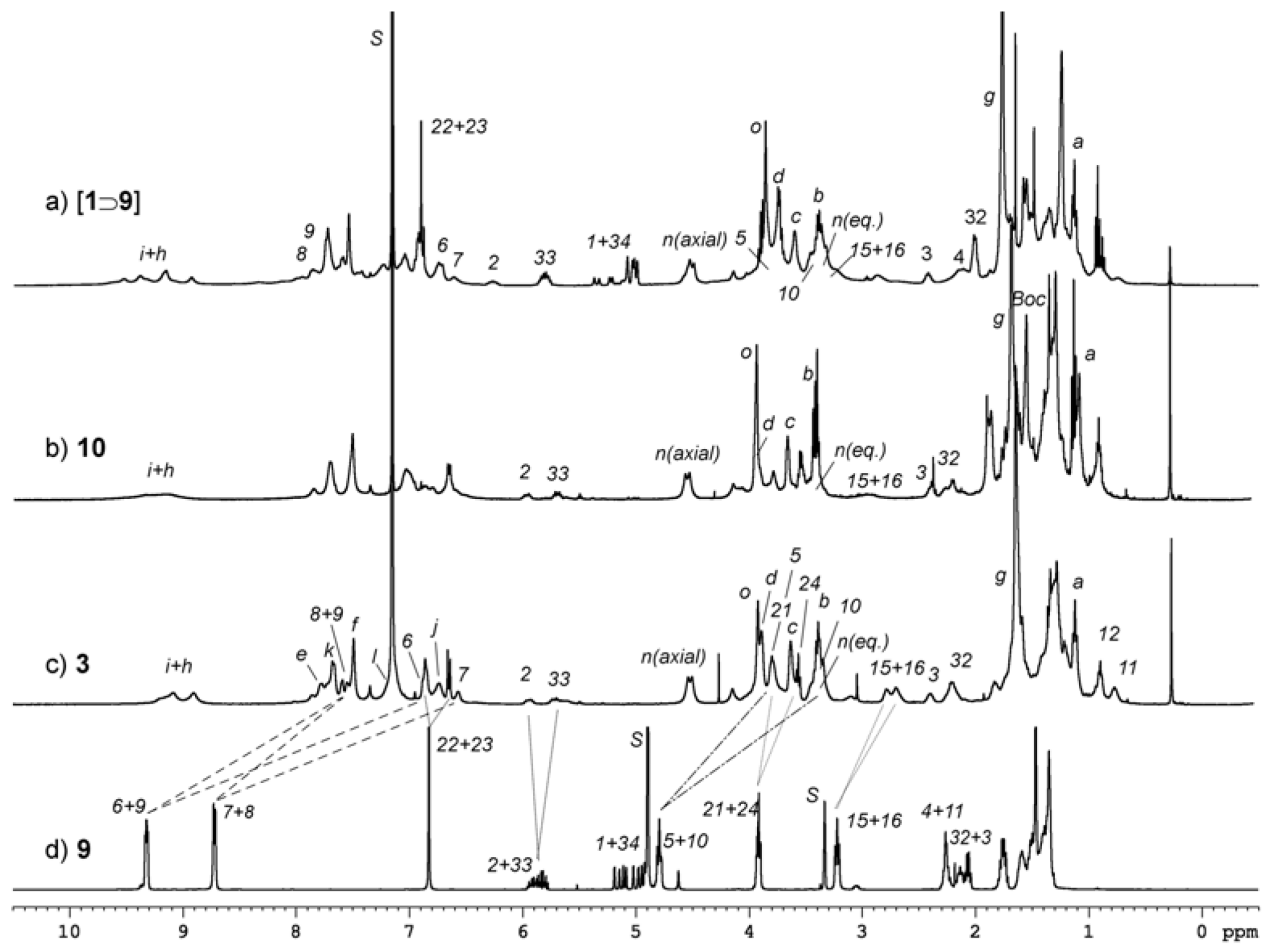
^1^H NMR stack plot (400 MHz) of pseudorotaxane [**1**⊃**9**]_up_ (**a**), [2]catenane **10** (**b**) and **3** (**c**) taken in benzene-d_6_. The spectrum of free axle **9** (**d**) was observed in methanol-d_4_ for solubility reasons. The labels on the signals indicate the assigned resonances for the calix[6]arene macrocycle **1** (letters) and for the open and closed thread **9** (numbers); see Scheme 2 for the labels assignment. The most important complexation induced shift (CIS) endured by the protons of thread **9** upon complexation and ring closure are indicated with dashed and dotted lines (note that the up-field shift endured by most of the signals of axle **9** in catenane **3** may also be ascribed to a change in the polarity of the solvent).

The ^1^H NMR spectrum of [**1**⊃**9**]_up_ (Figure 4a) shows, as a general feature, how all the resonances of the portion of **9** threaded into **1** are affected by the anisotropic shielding effect exerted by the electron-rich aromatic cavity. The consequent upfield shift was particularly marked for the protons belonging to the axle bipyridinium unit. These protons, labelled in Scheme 2 and Figure 4a from *9* to *6*, give rise to broad and partially overlapped resonances at *δ* = 7.7, 7.6, 6.6 and 6.7 ppm, in this order. Nevertheless, they were easily recognisable through the correlation with the chemical shift of their attached ^13^C nuclei. The latter are indeed less affected by the anisotropic effect of the aromatic cavity and originate very diagnostic cross-peaks in heterocorrelated 2D NMR experiments (see e.g., Figure S6 of the Supplementary Material). In comparison with their chemical shift in the free axle **9** (Figure 4d), these resonances experience upfield shifts from 0.9 (*8*) and 1.6 (*9*) up to 2.1 (*7)* and 2.6 (*6*) ppm. The higher shielding of protons *6* and *7* reflects the position of their pyridinium ring, that is more deeply engulfed in the calixarene cavity than the one bearing protons *8* and *9*. The same large shielding effect was endured by the resonances of the two methylene groups linked to the bipyridinium unit, labelled as *5* and *10* in Scheme 2, and occurring at *δ* = 3.9 and 3.4 ppm, respectively (Figure 4a). As noted before, these resonances are totally hidden by the large and broad signals of the calix[6]arene **1**. Nevertheless, they are found as recognizable cross-peaks in their ^1^H and ^13^C heterocorrelated NMR spectra, owing to their characteristic ^13^C chemical shift in the 59–60 ppm region (see Supplementary Material). In the region of the spectrum of [**1**⊃**9**]_up_ from 4.8 to 6.5 ppm, two different sets of signals ascribable to the vinyl group present on each chain are observed. The unequivocal attribution of other signals of the thread was not possible because of the higher fluxionality of the complex on the NMR time scale.

For the following catenation reaction, the key factors affecting the intramolecular cyclization, besides the size of the resulting macrocycles, are the concentration of the pre-formed pseudorotaxane and the molar ratio between the latter and the catalyst used to promote the methatesis reaction. The second generation Grubbs catalyst was added (15% moles) to a 1 mM solution of [**1**⊃**9**]_up_ in degassed dry toluene, in agreement with literature procedures [32,33,34]. After a two-day reaction at room temperature, the Boc-protected [2]catenane **10** (Scheme 2) was isolated at 17% yield after chromatographic separation. The A-station was finally obtained by treatment of **10** with trifluoroacetic acid, thus affording the target [2]catenane **3**.

The ^1^H NMR spectra of **10** and **3** (Figure 4b,c) confirm their identity. The most relevant evidence of the occurred intramolecular metathesis is the disappearance in both spectra of the signals of the vinyl methylene protons *1* and *34*, and the presence of two methyne signals at *δ* = 5.9 and 5.7 ppm, ascribed to the newly formed double bond (protons *2* and *33*). The multiplicity of these multiplets suggests that the RCM reaction has afforded both the *Z* and *E* geometrical isomers of the C=C bond. Moreover, in the TOCSY spectrum of **3** (see Figure S10 of the Supplementary Material), a series of correlations that links protons *2* to *32* can be evidenced. This series is also related with proton *33*, thus confirming that the intramolecular closure took place. The presence of a sharp singlet for the OCH_3_ protons at the lower rim of **1** in the spectra of **10** and **3** is consistent with the formation of a single orientational [2]catenane isomer [25,31]. The preference of the calixarene wheel for the B-station over the A-station in **3** is not unexpected in the light of previous results obtained on related pseudorotaxanes [35] and rotaxanes [36]. Such a behavior is most likely determined by the aromatic nature of the bipyridinium unit and its larger positive charge compared to the ammonium site. The intramolecular closure of the thread was also confirmed by high-resolution mass spectrometry measurements. The high-resolution mass spectrometry (HRMS) measurements carried out on catenane **10** (see Figure S8 of the Supplementary Material) shows almost exclusively a monoisotopic peak at *m*/*z* = 1103.6755 (*z* = 2) that was assigned to the doubly charged species. In addition to the disappearance of the singlet relative to the Boc group at *δ* = 1.55 ppm, the ^1^H NMR spectrum of **3** (Figure 4c), in comparison with that of **10** (Figure 4b), exhibits a better definition of the peaks that, together with the aid of 2D NMR experiments (see Figures S10–S12 of the Supplementary Material), allowed an almost complete assignment of the protons of the annulated thread. In particular, in the 2D ROESY NMR spectrum, several cross-peaks are present, which confirm the spatial proximity between the double bond of the annulated thread and the methoxy groups at the calix[6]arene lower rim (see Figure S12 of the Supplementary Material for further details).

### 2.2. Electrochemical Measurements

**Figure 5:**
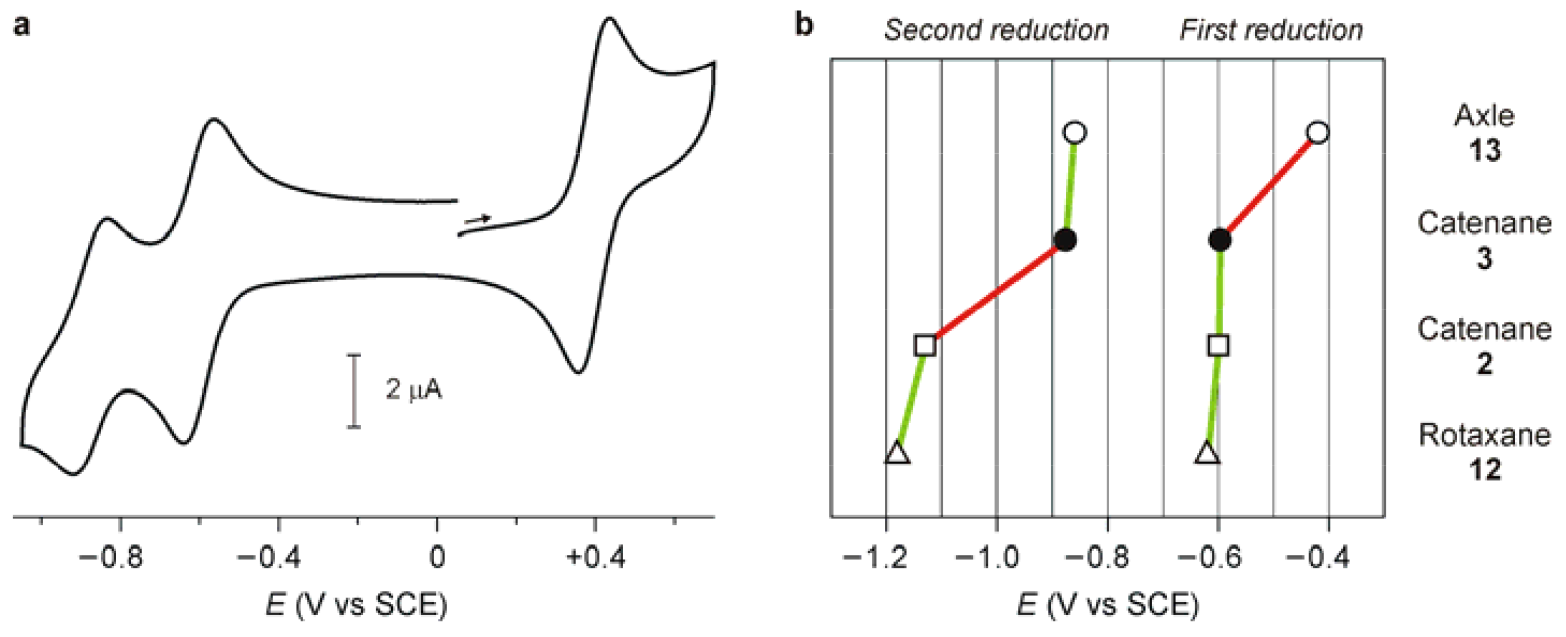
Electrochemical measurements: (**a**) Cyclic voltammogram of an argon-purged acetonitrile solution of catenane **3** (0.03 mM, 0.04 M tetraethylammonium hexafluorophosphate (TEAPF_6_); scan rate 200 mV s^−1^); the wave in the region of positive potentials is that of ferrocene used as an internal standard. (**b**) Genetic diagram for the first and second reduction processes for (from top to bottom): the free bipyridinium axle **13** (empty circles), the [2]catenane **3** (solid circles), the one-station [2]catenane **2** (squares); and the one-station [2]rotaxane **12** (triangles).

The electrochemical behavior of the oriented [2]catenane **3** in acetonitrile was investigated by cyclic and differential pulse voltammetry techniques. The results obtained were compared to those gained by measuring the electrochemical behavior of: (a) the symmetrical one-station [2]catenane **2** [25], (b) the one-station rotaxane **12** [37] and (c) the free 1,1′-dioctyl-4,4′-bipyridinium axle **13** [29] (Figure 2). Catenane **3** shows two reversible monoelectronic reduction processes at −0.597 and −0.877 V versus the standard calomel electrode (SCE) (Figure 5a), which were assigned to the first and second reduction of the bipyridinium unit, respectively. As illustrated in the genetic diagram depicted in Figure 5b, the first reduction potential of **3** is shifted to more negative values with respect to the same process in the bare axle **13**. We attributed this shift to the stabilization provided to the B-station of the ‘track’ ring by the π–rich cavity of **1**. Indeed, the shift measured for **3** is similar to that observed for the interlocked species **2** and **12**, in which the bipyridinium unit is confined inside the aromatic cavity of **1**. On the other hand, the value of the second reduction potential of **3** is comparable to that of free **13**. Such an observation suggests that the calixarene wheel does not interact any longer with the monoreduced bipyridinium site of the other ring, in contrast to what was observed for the one-station catenane **2** [25]. The large negative shift detected for the second reduction of rotaxane **12**, in which the short axle prevents the calixarene from moving far away from the reduced station, confirms that in the monoreduced catenane **3** the calixarene wheel is no longer encircling the B-station.

Taken together, these findings are consistent with a translational movement of the calixarene along the ‘track’ ring, which takes place as a consequence of the first one-electron reduction of **3**. Therefore, the presence of the dialkylammonium unit as a secondary recognition site capable of competing with the bipyridinium radical cation for the calixarene wheel is necessary to enable the electrochemically triggered co-conformational switching. The full reversibility of all the voltammetric waves confirms that no disassembly of the system takes place upon electrochemical stimulation; it also indicates that (i) the calixarene returns around the bipyridinium unit once the latter is reoxidized to the dicationic state, and (ii) the movements are fast on the time scale of the electrochemical experiment for all the employed scan rates.

## 3. Materials and Methods

### 3.1. Synthesis

Toluene, THF, acetonitrile and dichloromethane were dried by following standard procedures. Other reagents were of reagent grade quality, obtained from commercial sources and used without further purification. Chemical shifts are expressed in ppm using the residual solvent signal as internal reference. Compounds **1** [38], **2** [25], **4** [39], **5** [35], **12** [37] and **13** [29] were obtained following reported procedures.

*1-(pent-4-en-1-yl)-[4,4′-bipyridin]-1-ium bromide* (**6**): 5-Bromo-1-pentene (0.64 g, 4.3 mmol) and 4,4′-bipyridine (2.00 g, 12.8 mmol) were dissolved in dry acetonitrile (50 mL) and refluxed overnight. After removing the solvent under reduced pressure, the crude residue was triturated with ethyl acetate (4 × 25 mL) to obtain **6** as a sticky white solid (0.88 g, 68%). ^1^H NMR (400 MHz, CD_3_OD, δ): 2.2–2.3 (m, 4H), 4.72 (t, *J* = 7.1 Hz, 2H), 5.09 (br. d, *J* = 10 Hz, 1H), 5.15 (br. d, *J* = 17 Hz, 1H), 5.8–6.0 (m, 1H), 8.02 (d, *J* = 6.1 Hz, 2H), 8.55 (d, *J* = 6.4 Hz, 2H), 8.85 (d, *J* = 6.1 Hz, 2H), 9.15 (d, *J* = 6 Hz, 2H); ^13^C NMR (100 MHz, CD_3_OD, δ) 29.8, 30.0, 60.8, 115.5, 122.2, 125.8, 136.1, 142.2, 145.2, 150.4 ppm; Electrospray Ionization mass spectrometry (ESI-MS) (+): *m*/*z* (%) = 225 (100) 226 (20) [M]^+^; Anal. Calcd for C_15_H_17_BrN_2_: C, 59.03; H, 5.61; N, 9.18. Found: C, 58.64; H, 5.38; N, 9.45%.

*Tert-butyl N-{6-[4-(undec-10-en-1-yloxy)phenoxy]hexyl}carbamate* (**7**): To a solution of **4** (1.27 g, 4.8 mmol) in anhydrous DMF (100 mL), K_2_CO_3_ (2 g, 14.5 mmol) and **5** (1.98 g, 5.3 mmol) were added. The reaction mixture was stirred for 18 h at 80 °C. After cooling at room temperature, the reaction was quenched with water (100 mL) and extracted with ethyl acetate (3 × 100 mL). The separated organic phase was dried over Na_2_SO_4_ and evaporated under reduced pressure. Pure product **7** was isolated by column chromatography (*n*-hexane/acetone 8:2) as a pale yellow oily compound (1.29 g, 58%). ^1^H NMR (400 MHz, CDCl_3_, δ): 1.3–1.5 and 1.45 (m and s, 38H), 1.7–1.8 (m, 4H), 2.05 (q, *J* = 7 Hz, 2H), 3.11 (br. t, *J* = 6.3 Hz, 2H), 3.88 (t, *J* = 6.5 Hz, 4H), 4.70 (br. s, 1H), 4.93 (ddd, *J*_1_ = 10.1 Hz, = 10.1, *J*_2,3_ = 1.2 Hz, 1H), 5.0 (ddd, *J*_1_ = 17.1 and *J*_2_ = 1.44 Hz, 1H), 5.7–5.9 (m, 1H), 6.81 (s, 4H); ^13^C NMR (100 MHz, CDCl_3_, δ): 14.1, 22.7, 25.8, 26.1, 26.6, 28.4, 28.9, 29.1, 29.3, 29.4 (2 res.), 29.5, 30.1, 31.6, 33.8, 40.5, 68.4, 68.6, 78.9, 114.2, 115.4 (2 res.), 139.1, 153.1, 153.2, 156.0 ppm; ESI-MS(+): *m*/*z* (%) = 484 (100) 485 (30) [M + Na]^+^; Anal. Calcd for C_28_H_47_NO_4_: C, 72.84; H, 10.26; N, 3.03. Found: C, 72.91; H, 9.32; N, 3.05%.

*Tert-butyl N-(6-bromohexyl)-N-{6-[4-(undec-10-en-1-yloxy)phenoxy]hexyl}carbamate* (**8**): Compound **7** (0.50 g, 1.1 mmol) was dissolved in anhydrous DMF (50 mL) and cooled at 0 °C. NaH (0.20 g of 60% dispersion in mineral oil, 2.2 mmol) was slowly added and the reaction mixture was stirred for 1 h at room temperature under inert atmosphere. The yellow solution was then cooled again at 0 °C and 1,6-dibromohexane (0.79 g, 3.25 mmol) was slowly added. The mixture was stirred for 24 h at room temperature. The reaction was carefully quenched with water (40 mL) and extracted with ethyl acetate (3 × 50 mL). The separated organic phase was dried over Na_2_SO_4_ and evaporated under reduced pressure. The residue was purified by column chromatography (*n*-hexane/acetone 9:1) to obtain **8** as a colorless oil (0.34 g, 50%). ^1^H NMR (300 MHz, CDCl_3_, δ): 1.2–1.6 and 1.46 (m and s, 36H), 1.7–1.8 and 1.8–1.9 (2m, 8H), 2.05 (br. q, 2H), 3.2 (br. s, 4H), 3.41 (t, *J* = 7 Hz, 2H), 3.90 (t, *J* = 6 Hz, 4H), 4.9–5.1 (m, 2H), 5.7–5.9 (m, 1H), 6.82 (s, 4 H); ^13^C NMR (100 MHz, CDCl_3_, δ): 25.9, 26.1, 26.7, 27.9, 28.3, 28.4, 28.5, 28.9, 29.1, 29.4 (2 res.), 29.5, 32.7, 33.8, 46.9, 47.0, 68.4, 68.6, 79.1, 114.1, 115.4 (2 res.), 139.2, 153.1, 153.2, 155.6 ppm; ESI-MS(+): *m*/*z* (%) = 624 (95) 625 (40) 626 (100) 627 (38) [M + H]^+^. Anal. Calcd for C_34_H_58_BrNO_4_: C, 65.37; H, 9.36; N, 2.24. Found: C, 66.11; H, 9.82; N, 1.98%.

*1′-(6-{[(tert-butoxy)carbonyl]({6-[4-(undec-10-en-1-yloxy)phenoxy]hexyl})amino}hexyl)-1-(pent-4-en-1-yl)-[4,4′-bipyridine]-1,1′-diium dibromide* (**9**): In a sealed glass reactor, salt 6 (0.11 g, 0.36 mmol) and bromide 8 (0.34 g, 0.55 mmol) were dissolved in 10 mL of dry acetonitrile and refluxed for 7 days. After cooling at room temperature, the solvent was evaporated under reduced pressure and the residue was dissolved in ethyl acetate (20 mL). The solution was cooled at 0 °C and the precipitation of the product was observed. The yellow solid was collected by Buchner filtration. Axle 7 was obtained as a yellow solid (0.19 g, 55%). Melting point (mp) > 300 °C dec.; ^1^H NMR (300 MHz, CD_3_OD, δ): 1.3–1.7 and 1.35 and 1.47 (m and 2s, 33H), 1.7–1.8 (m, 4H), 2.06 (br. q, 2H), 2.13 (br. t, 2H), 2.2–2.3 (m, 4H), 3.22 (t, *J* = 7.3 Hz, 4H), 3.8–3.9 (m, 4H), 4.79 (t, *J* = 6.8 Hz, 4H), 4.93 (ddd, *J*_1_ = 10 Hz, *J*_2,3_ = 1.4 Hz, 1H), 4.99 (ddd, *J*_1_ = 17 Hz, *J*_2,3_ = 1.4 Hz, 1H), 5.09 (ddd, *J* = 10.1, *J*_2,3_ = 1.3 Hz 1H), 5.16 (ddd, *J*_1_ = 17 Hz, *J*_2,3_ = 1.3 Hz, 1H), 5.8–6.0 (m, 2H), 6.82 (s, 4H), 8.72 (d, *J* = 6.4 Hz, 4H), 9.3–9.4 (m, 4H); ^13^C NMR (100 MHz, CD_3_OD, δ): 25.6, 25.8, 26.3, 27.4, 28.7, 28.8, 29.1 (3 res.), 29.3, 29.8, 30.1, 31.1, 33.5, 48.2, 61.4, 61.8, 68.1, 68.3, 79.3, 113.3, 115.1, 115.6, 127.0, 136.1, 138.8, 145.7, 149.9 (2 res.), 153.2, 153.3, 156.0 ppm. ESI-MS(+): *m*/*z* (%) = 769 (100) 770 (55) [M-H]^+^, 669 (100) 670 (55) [M-H-Boc]^+^; Anal. Calcd for C_49_H_75_Br_2_N_3_O_4_: C, 63.29; H, 8.13; N, 4.52. Found: C, 62.91; H, 8.02; N, 4.81%.

*[2]catenanes* (**3**) *and* (**10**): In a two-neck flask under inert atmosphere, viologen salt **9** (0.07 g, 0.08 mmol) was suspended in a solution of wheel **1** (0.21 g, 0.08 mmol) in dry toluene (80 mL, concentration = 0.001 M). The suspension was stirred at room temperature for one day, until the solution turned dark red and the salt was completely dissolved. Argon was then bubbled into the system for 30′, and 2nd gen. Grubbs catalyst (5 mg) was added. The reaction mixture was stirred at room temperature under bubbling argon and monitored by thin-layer chromatography (TLC). After 3 days, the solvent was removed and the residue was portioned between dichloromethane and water. The separated organic phase was evaporated under reduced pressure. Purification of the residue by column chromatography (dichloromethane/methanol 95:5) yielded catenane **10** as a red solid compound. mp. > 300 °C dec; ^1^H NMR (400 MHz, C_6_D_6_, δ): 0.9, 1.1, 1.13, 1.2–1.5, 1.5–1.9, 1.54 and 1.68 (br. s., br. s., t, m, m, s and s), 2.2 (br. s, 2H), 2.3 (br. s, 2H), 2.9 (br. s, 2H), 3.4 (br. s, 6H), 3.9 (br. s, 9H), 4.6 (br. s., 6H), 5.71 (br. s, 1H), 6.0 (br. s, 1H), 6.9 (br. s, 4H), 6.5–8.0 (m, 8H); ^13^C NMR (100 MHz, C_6_D_6_, δ): 14.0, 15.2, 22.8, 26.1, 26.2, 26.3, 26.7, 26.9, 28.4, 28.8, 29.5, 29.7, 29.9, 31.3, 31.6, 32.0, 32.5, 33.1, 34.6, 34.8, 35.5, 38.1, 59.7, 61.1, 62.3, 66.4, 67.9, 68.5, 70.0, 72.6, 78.0, 114.9, 115.5, 116.4, 117.6, 121.2, 124.2, 128.9, 129.3, 136.8, 140.7, 143.1, 148.3, 152.6 ppm; HRMS (*m/z*): [M]^2+^ calcd for C_137_H_179_N_9_O_16_, 1103.1729; found, 1103.1743.

The isolated **10** was then dissolved in 10 mL of anhydrous dichloromethane and 5 mL of trifluoroacetic acid was added dropwise. After stirring for 2 h at room temperature, the solvent was removed under reduced pressure, to afford catenane **3** as a red solid compound (0.03 g, 17% of overall yield starting from **9**). ^1^H NMR (400 MHz, C_6_D_6_, δ): 0.8 (br. s, 2H), 0.9 (br. t, 9H), 1.1–1.7 and 1.65 (m and s, 61H), 1.8 (br. s, 2H), 2.2 (br. s, 2H), 2.4 (br. s, 4H), 2.7 and 2.8 (2 br. s, 2H), 3.1 (br. s, 2H), 3.4 (br. s, 6H), 3.6 (br. s., 8H), 3.8 (br. s, 10H), 3.9 (br. s, 6H), 3.93 (s, 9H), 4.45 (d, *J* = 11,6 Hz, 6H), 5.7 (br. s, 1H), 5.9 (br. s, 1H), 6.6 (br. s, 2H), 6.61 (d, *J* = 8.0 Hz, 4H), 6.7 and 6.8 (2br. s, 8H), 7.5 and 7.6 (3 br. s, 16H), 8.8–9.2 (m, 7 H); ^13^C NMR (100 MHz, C_6_D_6_, δ): 14.1, 15.2, 22.8, 24.2, 24.7, 25.5, 26.1 (2 res.), 26.5, 28.9, 29.0, 29.2, 29.4, 29.6, 29.8 (2 res.), 29.9, 31.3, 31.5, 31.6, 32.0, 32.6, 32.9, 34.5, 38.2, 47.1, 47.6, 59.7, 60.6, 61.1, 62.3, 66.3, 68.0 (2 res.), 69.9, 72.5, 115.1, 115.5, 116.3, 116.5, 117.8, 119.2, 121.4, 124.6, 125.6, 127.0, 128.3, 129.0, 129.2, 129.6, 131.5, 132.1, 134.1, 136.8, 140.6, 143.2, 143.9, 148.2, 152.7, 153.0, 153.4, 153.9, 161.7, 162.0, 162.4, 162.7 ppm.

### 3.2. Electrochemical Measurements

Cyclic voltammetric experiments were carried out at room temperature in argon-purged acetonitrile (romil Hi-dry) with an Autolab 30 multipurpose instrument (Eco Chemie, Utrecht, The Netherlands) interfaced to a PC. The working electrode was a glassy carbon electrode (0.07 cm^2^); its surface was routinely polished with a 0.3 μm alumina–water slurry on a felt surface. The counter electrode was a Pt wire, separated from the solution by a frit; an Ag wire was employed as a quasi-reference electrode, and ferrocene (Fc) was present as an internal standard (E(Fc^+^/Fc) = +0.395 V vs. SCE). Fc was added from a concentrated acetonitrile solution (typically 0.1 M). The concentration of the compound examined was in the range 2 × 10^−4^ M–4 × 10^−4^ M and tetraethylammonium hexafluorophosphate (TEAPF_6_) 0.04 M was added as supporting electrolyte. Cyclic voltammograms (CV) were obtained at sweep rates varying typically from 0.1 to 1 V s^−1^. In any instance, the full electrochemical reversibility of the voltammetric wave of Fc was taken as an indicator of the absence of uncompensated resistance effects. Differential pulse voltammograms (DPV) were performed with a scan rate of 20 mV s^−1^, a pulse height of 75 mV, and a duration of 40 ms. For reversible processes, the same halfwave potential values were obtained from the DPV peaks and from an average of the cathodic and anodic CV peaks.

### 3.3. Molecular Modelling

Molecular mechanics calculations to model the structure of catenane **3** were carried out in vacuo using the MMFF94s force field [40] implemented in the Avogadro (ver. 1.2) molecular modelling software [41]. The rendering of the structures was obtained with the Mercury software (Ver. 3.10, The Cambridge Crystallographic Data Center, Cambridge, UK) [42].

## 4. Conclusions

We have described the design, synthesis and properties of an electroactive [2]catenane in which a calix[6]arene wheel is interlocked with a non-symmetric oriented ‘track’ ring that contains two recognition sites. The synthetic strategy involves a ring closing metathesis reaction performed on an oriented pseudorotaxane, self-assembled by taking advantage of the directional threading of the calixarene by non-symmetric bipyridinium axles.

The results of voltammetric experiments performed on the novel catenane, together with comparison with the behavior of previously investigated parent compounds, show that the calixarene encircles the dicationic bipyridinium unit, and moves away from it upon one-electron reduction. The reversibility of all the voltammetric processes highlights the stability of the catenane under the conditions employed, and indicates that the electrochemically triggered movements are fast and reversible.

At present, we are unable to distinguish between the two non-equivalent rotation directions, as depicted in Figure 1b, and to tell whether one of the two is preferred. However, one path linking the bipyridinium and ammonium stations on the ‘track’ ring (six methylene units) is significantly shorter and less hindered than the other (a chain of 26 atoms that includes a phenyl moiety), and it can be reasonably hypothesized that the shorter one will also be the faster. A deeper insight into these issues can be provided by the study of the opposite orientational isomer of the catenane, whose synthesis is currently underway in our laboratory.

## Supplementary Materials

The following items are available online. Figures S1 and S2: NMR characterization of axle **9**, Figures S3–S7: NMR characterization of pseudorotaxane [**1**⊃**9**]_up_, Figures S8–S9: NMR and HRMS characterization of [2]catenane **10**, Figures S10–S14: NMR and electrochemical characterization of [2]catenane **3**.
