# Supplementary material for: Electrochemically Triggered Co-Conformational Switching in a [2]catenane Comprising a Non-Symmetric Calix[6]arene Wheel and a Two-Station Oriented Macrocycle
Source: Molecules. 2018 May 11;23(5):1156. doi: 10.3390/molecules23051156 (PMC6100520; doi:10.3390/molecules23051156)
Supplement: Supplementary file 1 [file molecules-23-01156-s001.pdf]

Electrochemically triggered co-conformational switching in a [2]catenane comprising a non-symmetric calix[6]arene wheel and a two-station oriented macrocycle

Valeria Zanichelli[1], Luca Dallacasagrande[1], Arturo Arduini[1], Andrea Secchi[1,*], Giulio Ragazzon[2,3], Serena Silvi[2,4] and Alberto Credi[4,5,6,*]

[1] Dipartimento di Scienze Chimiche, della Vita e della Sostenibilità Ambientale, Università di Parma, Parco Area delle Scienze 17/A, 43124 Parma, Italy. andrea.secchi@unipr.it

[2] Dipartimento di Chimica "G. Ciamician", Università di Bologna, Via Selmi 2, 40126, Italy.

[3] Current address: Dipartimento di Scienze Chimiche, Università di Padova, via Marzolo 1, 35131 Padova, Italy.

[4] Center for Light Activated Nanostructures (CLAN), Università di Bologna and Consiglio Nazionale delle Ricerche, via Gobetti 101, 40129 Bologna, Italy. alberto.credi@unibo.it

[5] Dipartimento di Scienze e Tecnologie Agro-alimentari, Università di Bologna, viale Fanin 50, 40127 Bologna, Italy.

[6] Istituto per la Sintesi Organica e la Fotoreattività, Consiglio Nazionale delle Ricerche, via Gobetti 101, 40129 Bologna, Italy.

Supplementary Material

Table of Contents

NMR characterization of axle **9** 2-3

NMR characterization of pseudorotaxane [**1**⊃**9**]$_{up}$ 4-7

NMR and HR-MS characterization of [2]catenane **10** 8-9

NMR and electrochemical characterization of [2]catenane **3** 10-14

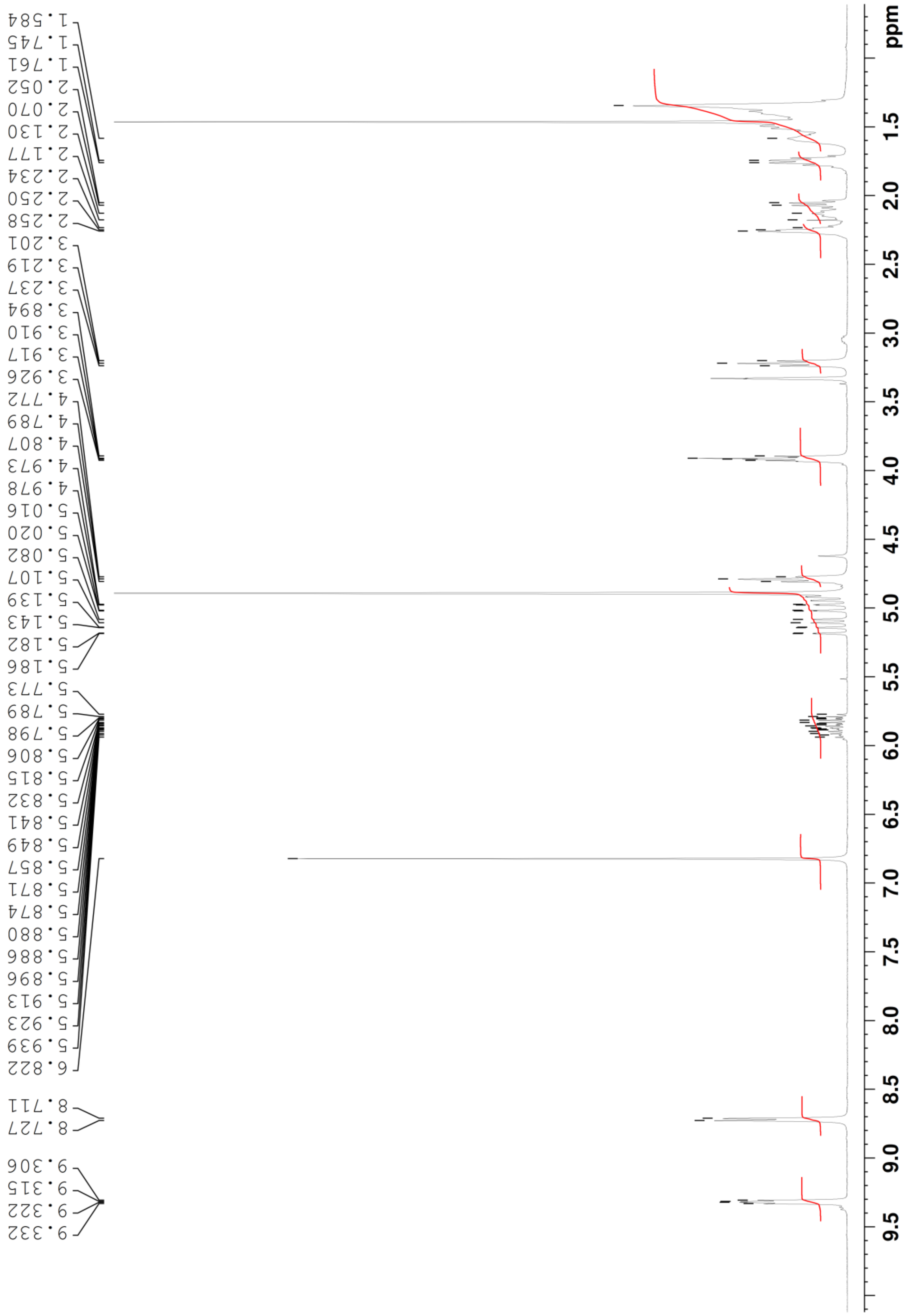

Figure S1. ^{1}H NMR spectrum (300 MHz, CD_3OD) of axle **9**.

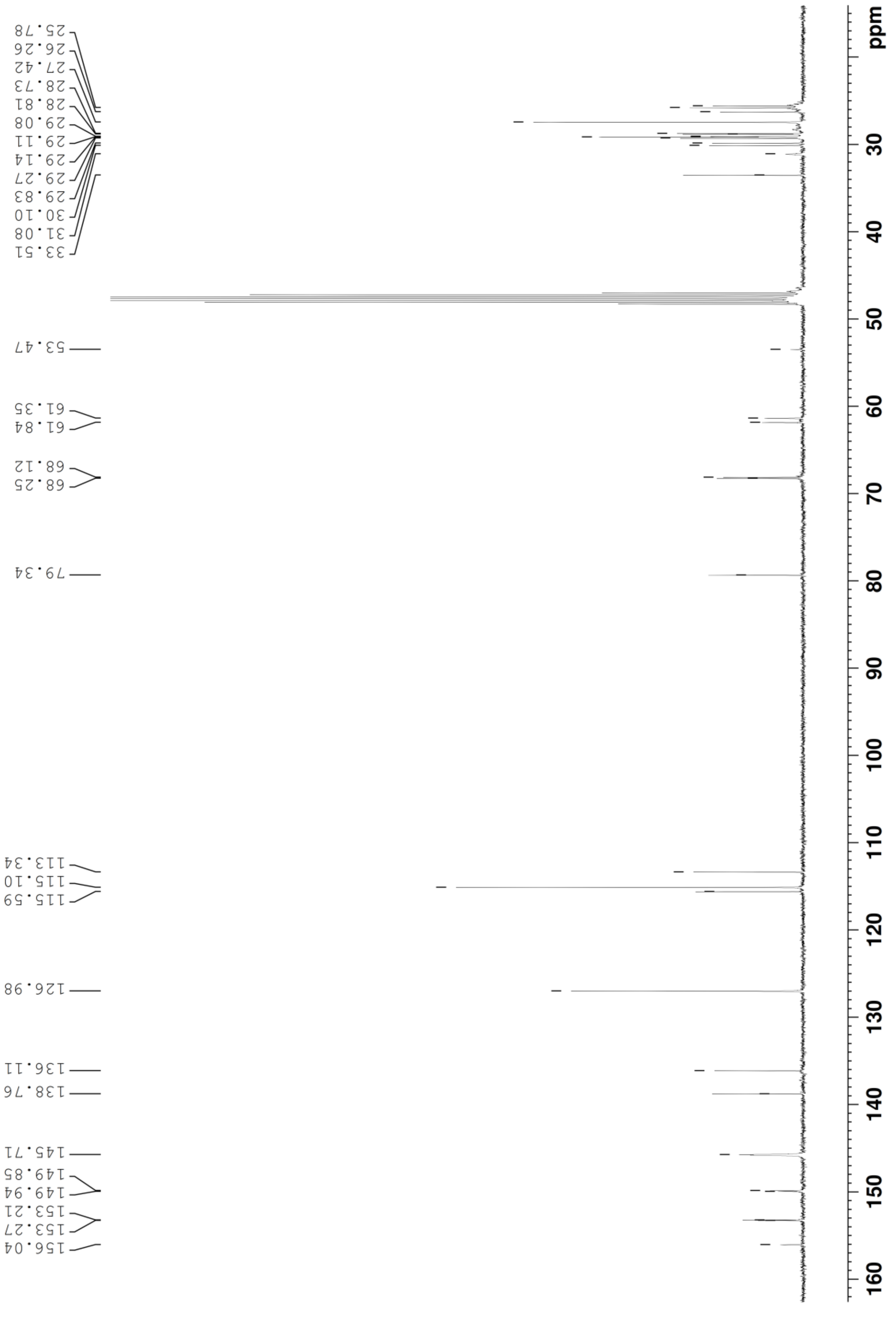

Figure S2. ^{13}C NMR spectrum (100 MHz, CD_3OD) of axle **9**.

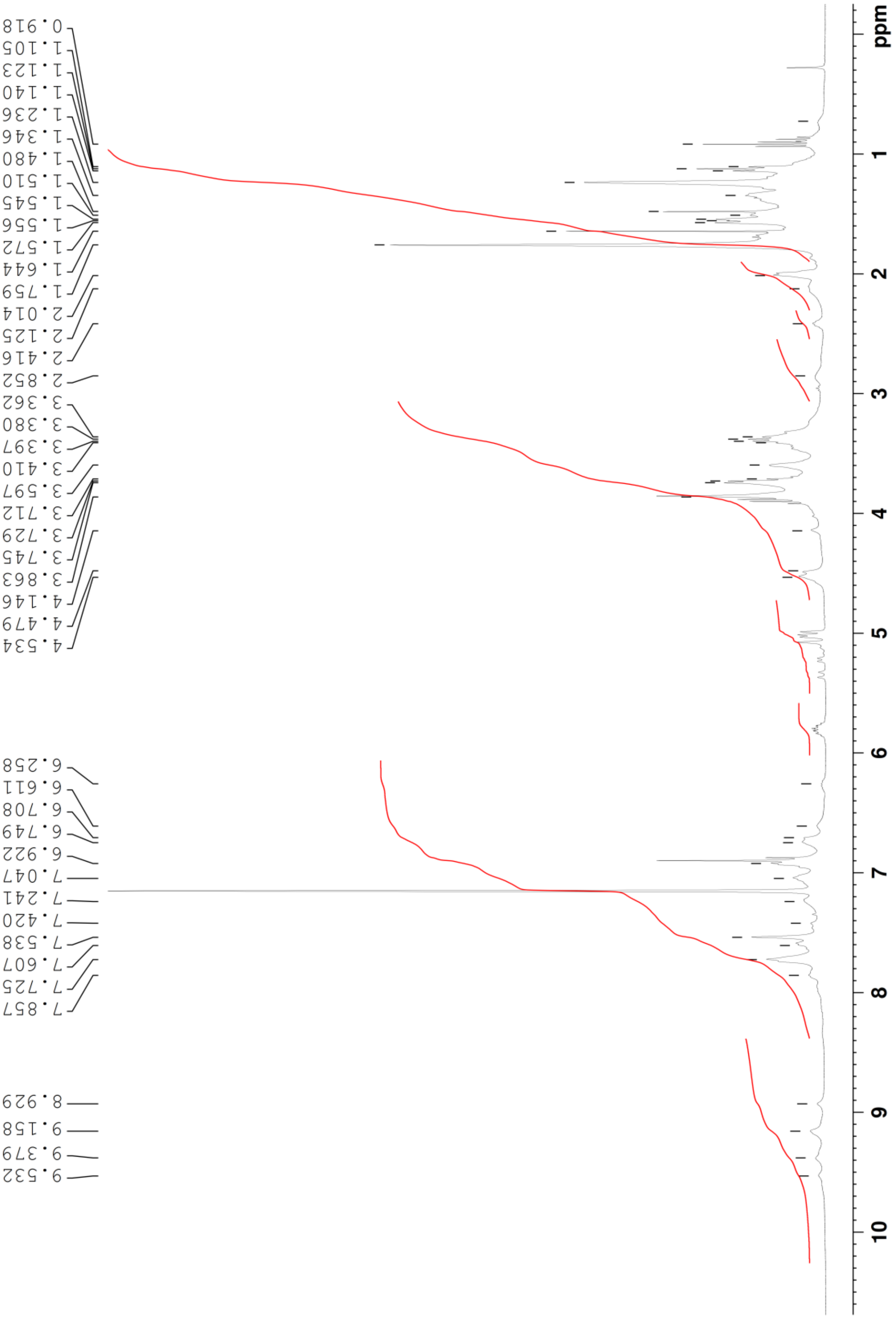

Figure S3. ^{1}H NMR spectrum (400 MHz, C_6D_6) of pseudorotaxane [**1**⊃**9**]$_{up}$.

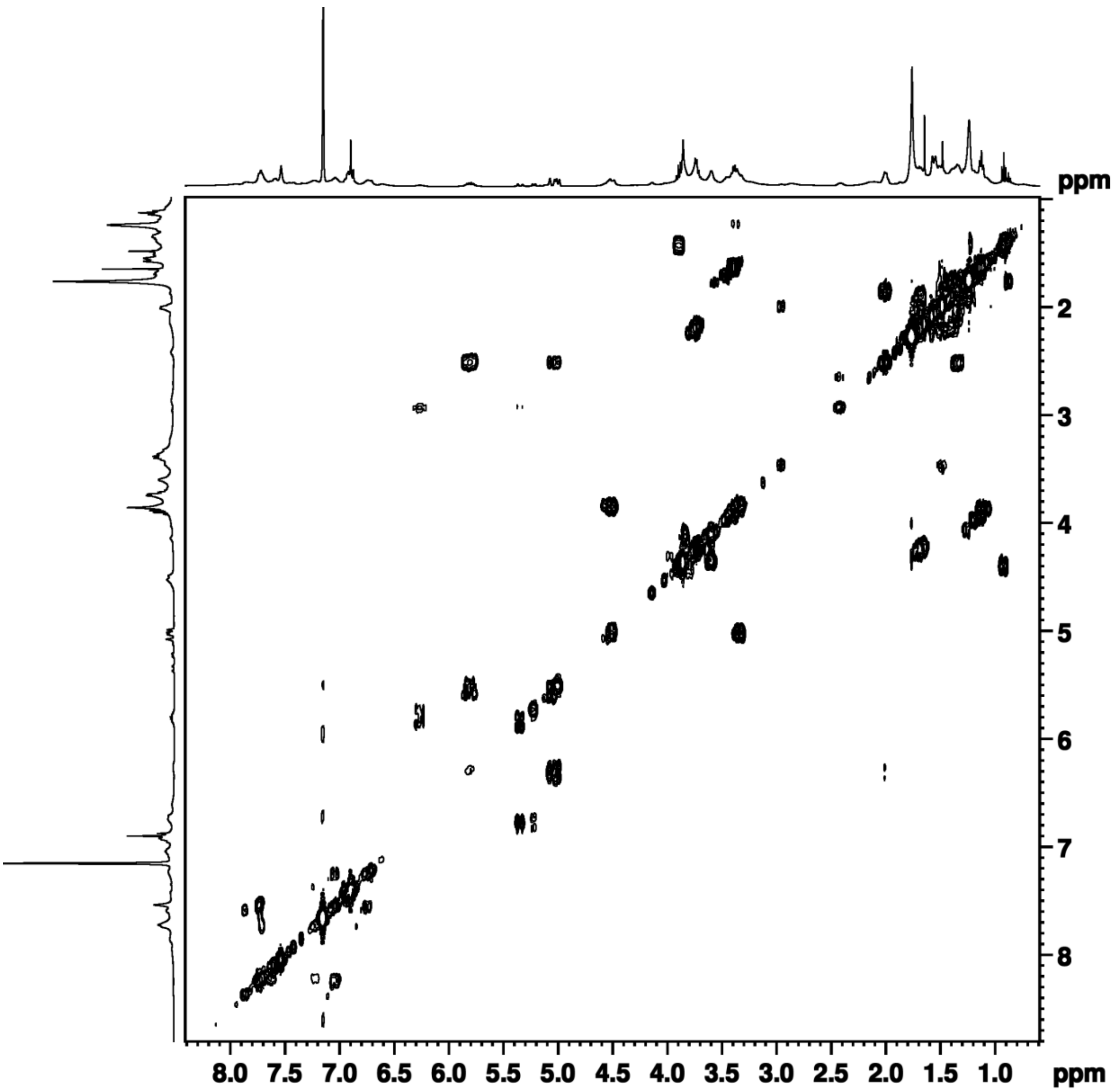

Figure S4. 2D COSY (top) spectrum (400 MHz, C_6D_6) of pseudorotaxane [**1**⊃**9**]$_{up}$

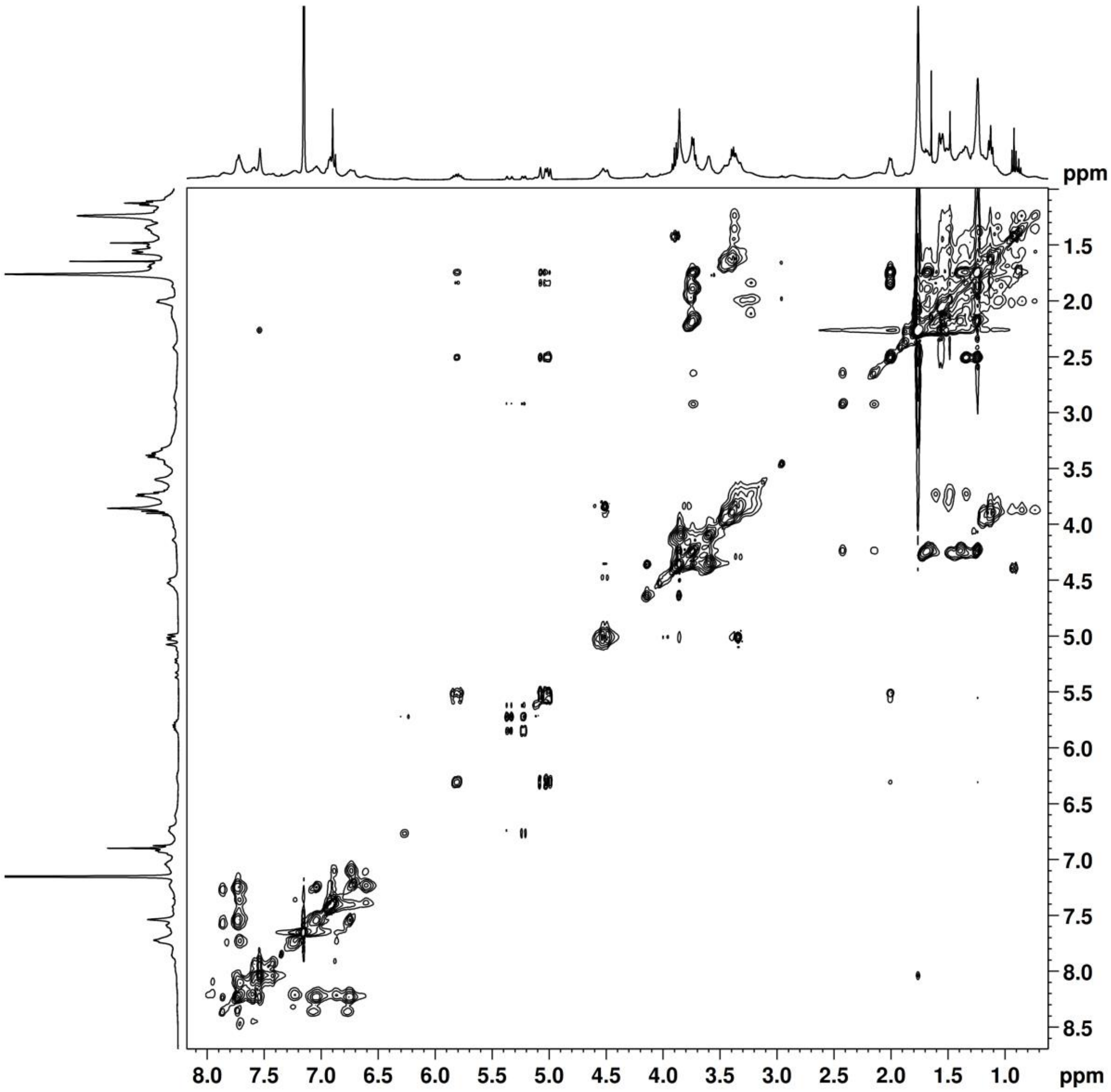

Figure S5. 2D TOCSY (MT = 0.08 s) spectrum (400 MHz, C_6D_6) of pseudorotaxane [**1**⊃**9**]$_{up}$.

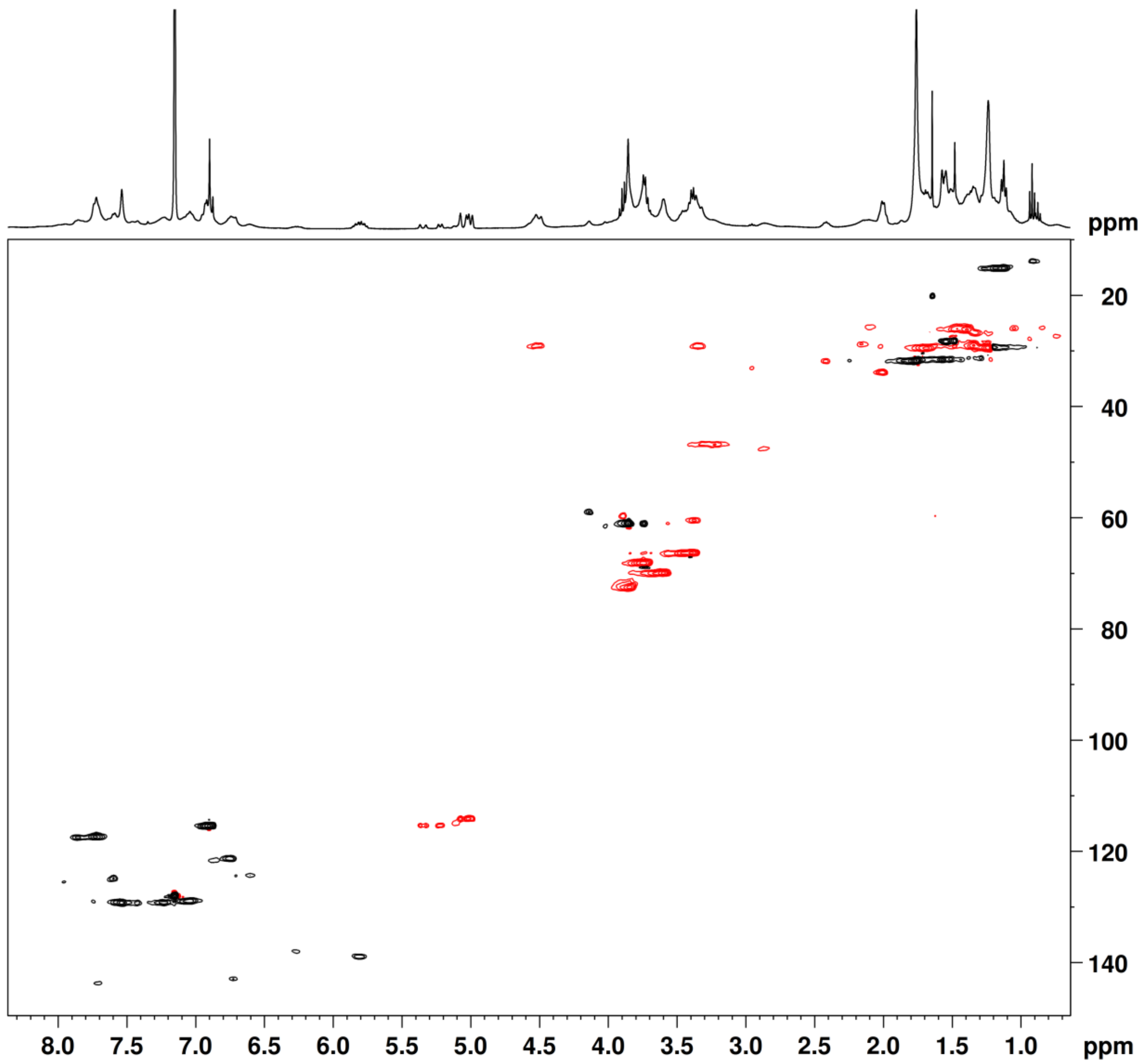

Figure S6. Edited 2D HSQC spectrum (400 MHz, C_6D_6) of pseudorotaxane [**1**⊃**9**]$_{up}$

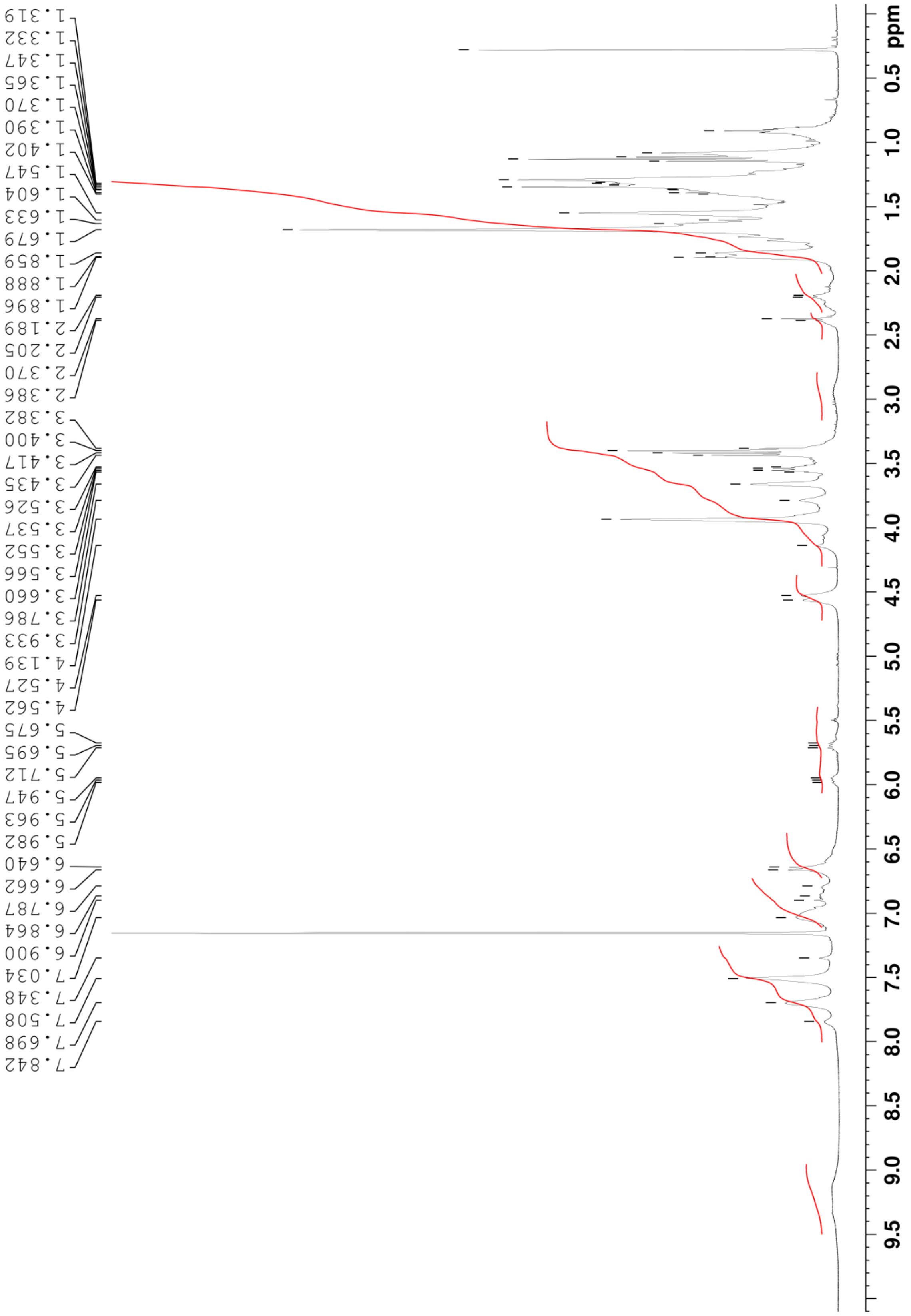

Figure S7. ^{1}H NMR spectrum (400 MHz, C_6D_6) of [2]catenane **10**

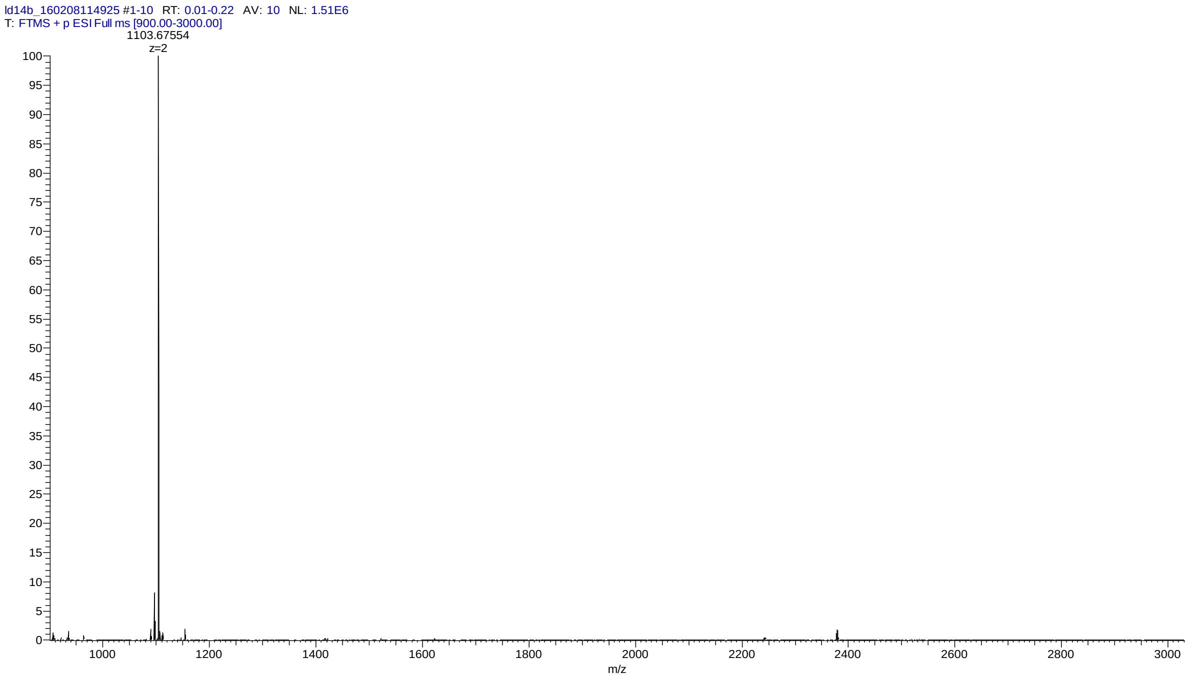

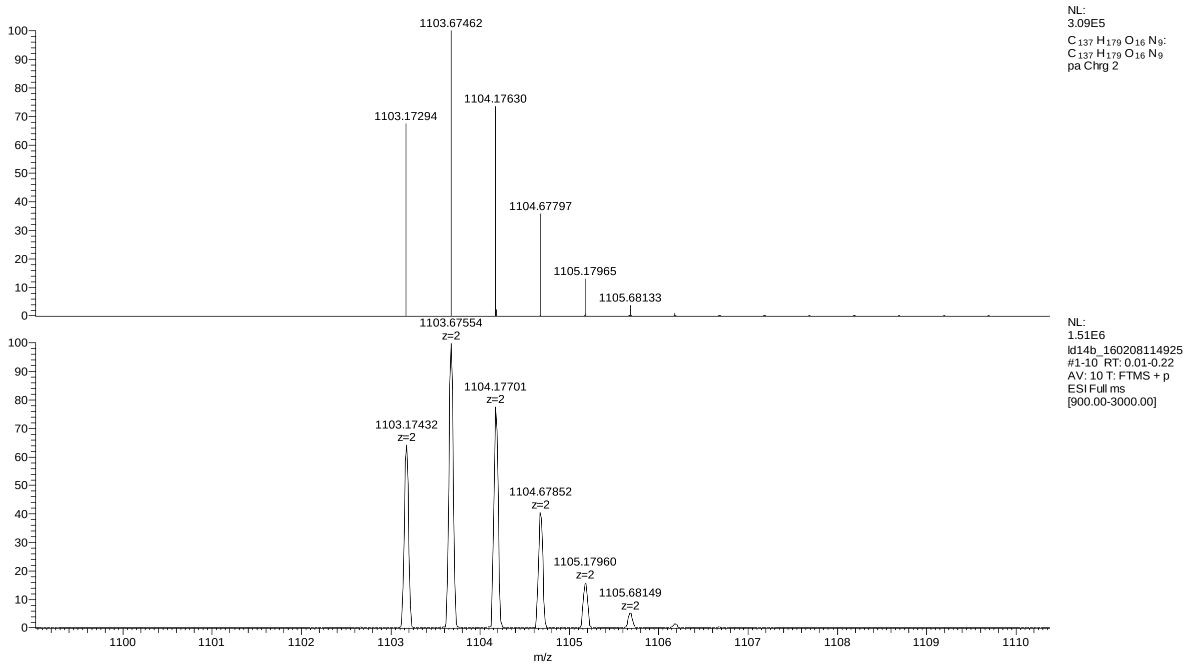

Figure S8. HR-MS (Orbitrap-LQ) of [2]catenane **10** (top) and comparison between the calculated and experimental isotopic distribution for the doubly charged specie (bottom).

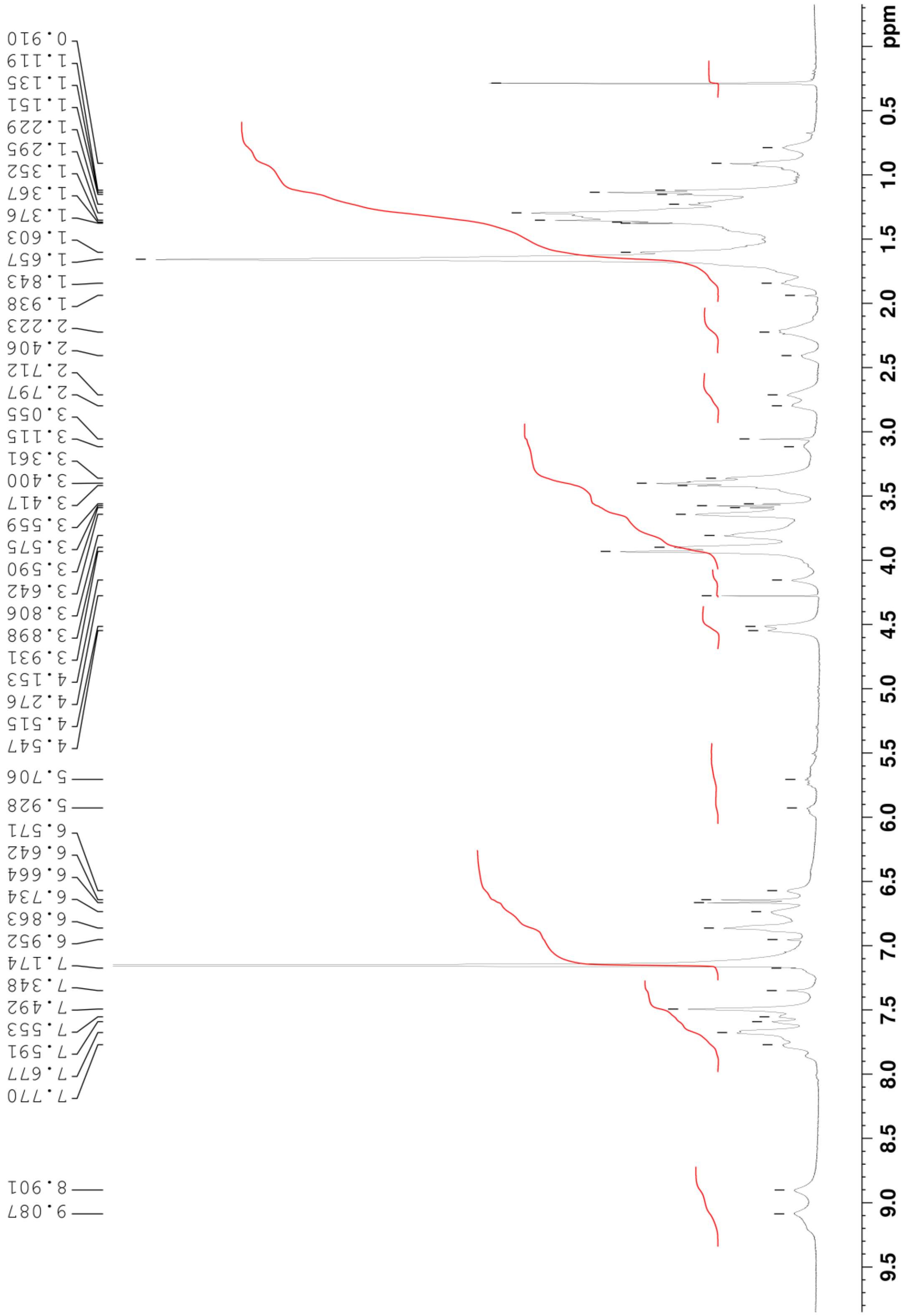

Figure S9. ^{1}H NMR spectrum (400 MHz, C_6D_6) of [2]catenane **3**

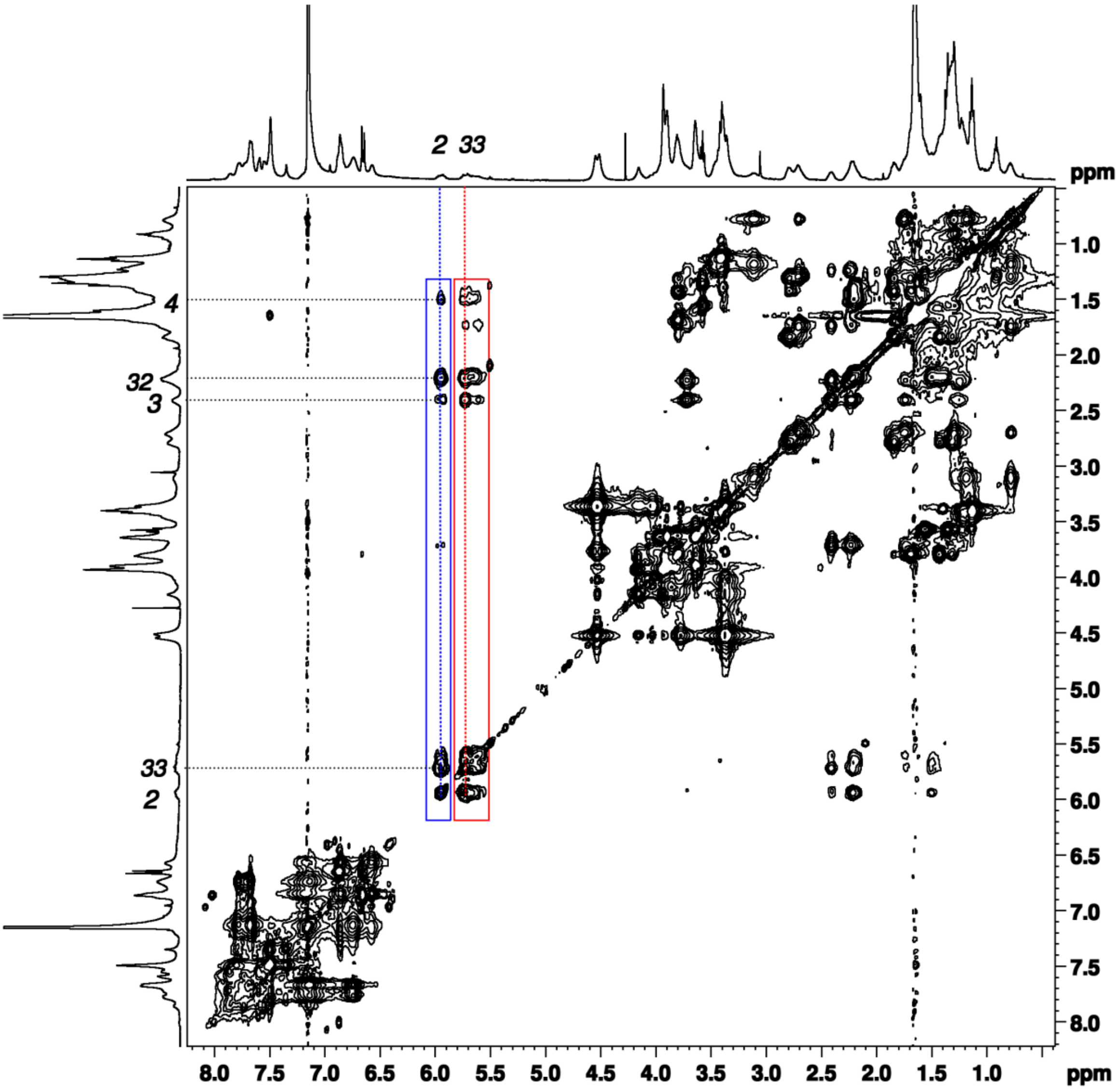

Figure S10. 2D TOCSY (MT = 0.04 s) spectrum (400 MHz, C_6D_6) of [2]catenane **3**. The scalar correlations linking proton *2* to proton *32* have been enclosed in a blue box. These series are also related with proton *33* (red box).

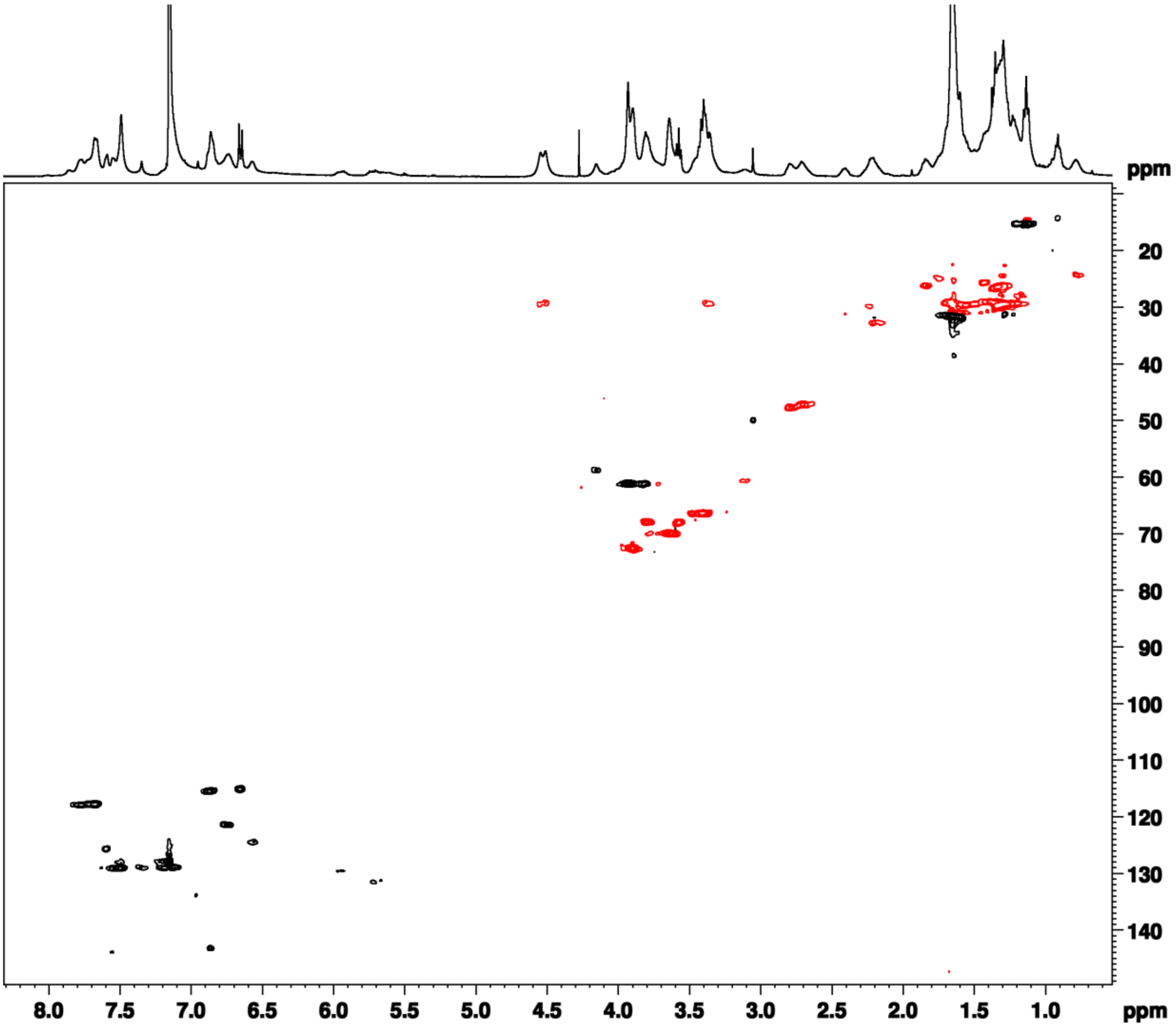

Figure S11. Edited 2D HSQC spectrum (400 MHz, C_6D_6) of [2]catenane **3**

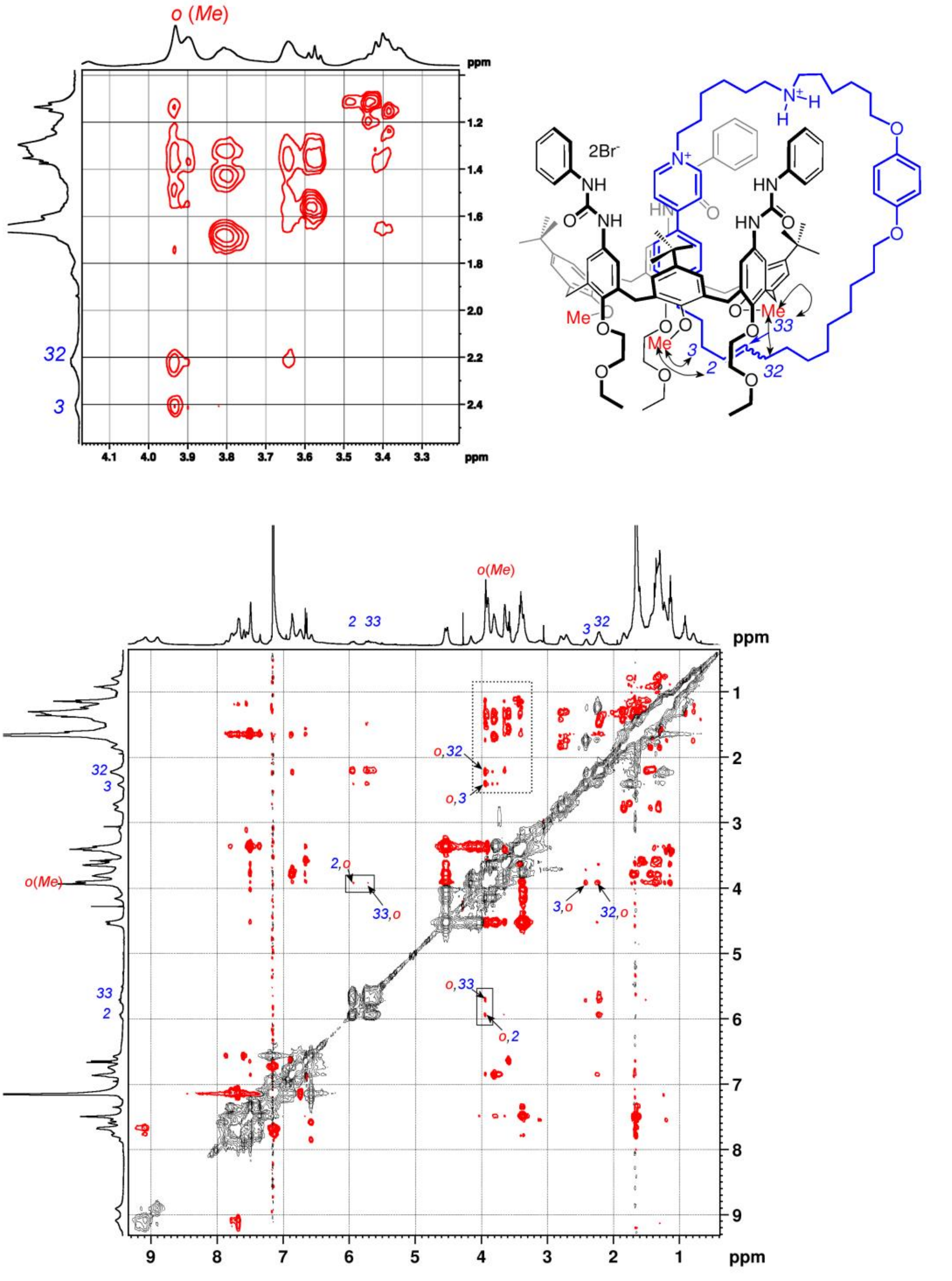

Figure S12. (Bottom) 2D ROESY spectrum (400 MHz, C_6D_6, SpinLock = 200 ms) of [2]catenane **3**. The cross-peaks showing the spatial proximity of protons *2*,*3*, *32* and *33* with the methoxy groups at the calix[6]arene lower rim have been indicated with arrows. (Top, left) Inset of the ROESY spectrum enlightening the cross-peaks generated by the dipolar interaction between the protons of the methoxy groups and the allylic protons 3 and 32 of the annulated thread of **3**. (Top, right) Molecular sketch showing the possible dipolar interaction (double arrows) at the calix[6]arene lower rim.

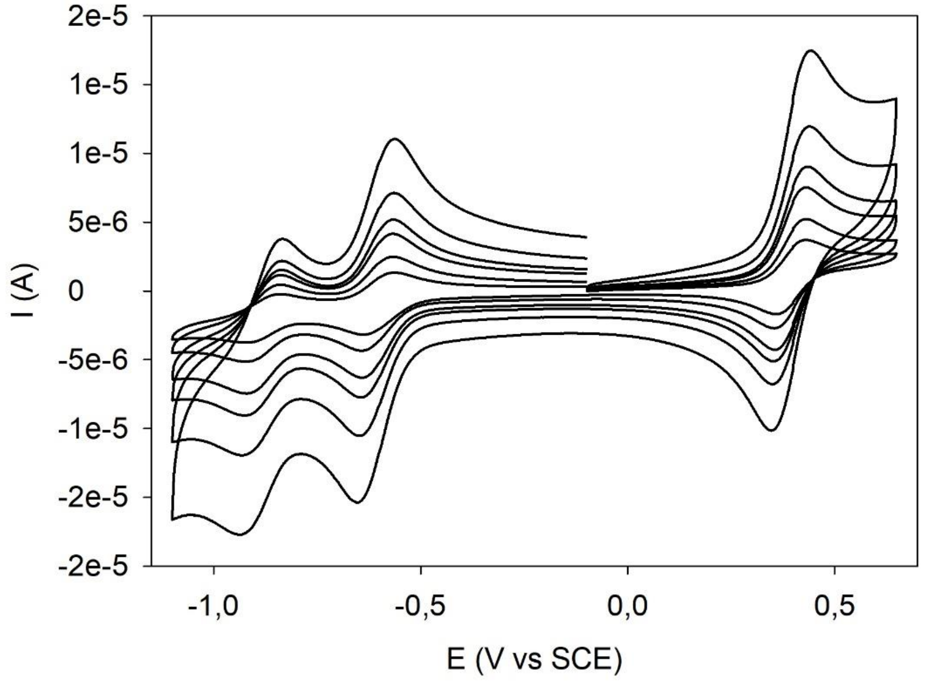

Figure S13. Cyclic voltammetric patterns of [2]catenane **3** at different scan rates (from 50 to 1000 mV s^{-1}). Conditions: argon-purged acetonitrile, 0.03 mM, 0.4 M $TEAPF_6$, glassy carbon electrode, r.t. The process at +0.395 V vs SCE is that of the ferrocene used as an internal standard.

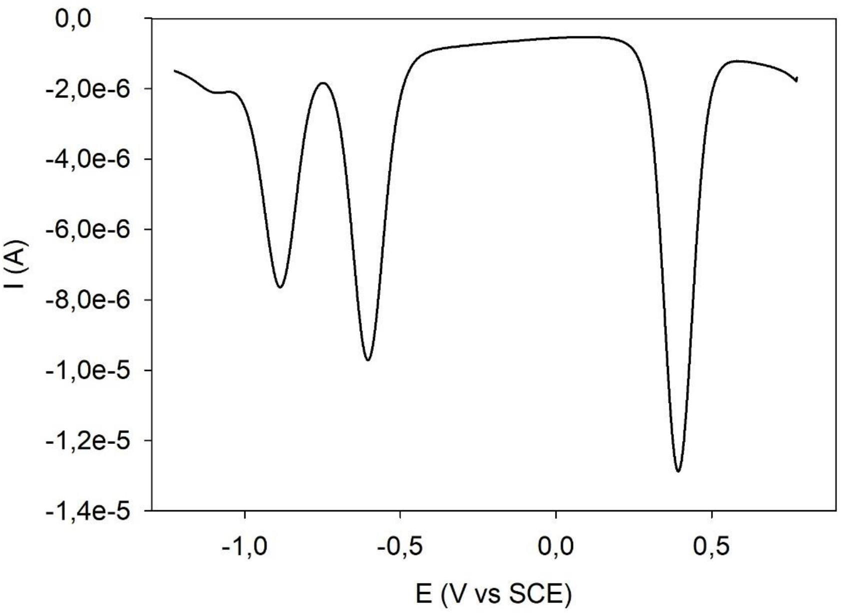

Figure S14. Differential pulse voltammogram of [2]catenane **3**. Conditions: argon-purged acetonitrile, 0.03 mM, 0.4 M $TEAPF_6$, glassy carbon electrode, r.t.; scan rate 20 mV s^{-1}, pulse height 75 mV, pulse duration 40 ms. The process at +0.395 V vs SCE is that of the ferrocene used as an internal standard.
